# Current state: Assessing DNA results considering transfer

**DOI:** 10.1016/j.fsisyn.2026.100733

**Published:** 2026-09-16

**Authors:** Jarrah R. Kennedy, Christophe Champod

**Affiliations:** a Texas Forensic Science Commission, Austin, TX, 78701, USA; b Faculty of Law, Criminal Justice and Public Administration, School of Criminal Justice, University of Lausanne, Batochime, CH-1015 Lausanne-Dorigny, Switzerland

**Keywords:** Activity level propositions, DNA-TPPR, Principles of evidence interpretation, Reporting, Testimony, Qualitative, Quantitative

## Abstract

This paper presents the current state of affairs regarding how DNA scientists consider the meaning of their results in light of questions about transfer and persistence—more formally termed evaluation of forensic observations given activity-level propositions. There are varying approaches both in the United States and abroad considering the assessment of DNA results in light of alleged activities. The authors distill the variability into: 1) ***how to interpret***: the methods used, the data considered, and whether qualitative or quantitative and 2) ***when to interpret***: through formal reporting in advance of trial or once questions arise during testimony. A selection of scientific and legal hurdles is discussed closing with suggestions for a potential path forward with an emphasis on defining high-risk case scenarios and adapting education opportunities in the United States.

## Highlights

• Discussion of the role of the DNA scientist beyond questions of sources.
• Are ethical obligations to communicate the limitations of DNA comparisons *in isolation* enough to prevent misunderstandings of the DNA results when it regards disputed activities?
• Interpretation of the DNA results in the wider context of the case is rarely addressed in a report.
• Waiting until testimony to provide opinions about DNA results considering transfer has led to a variety of approaches—some more scientifically justifiable than others—but all have associated risks.
• Education of both scientists and the criminal justice community is the key to forging a path forward together.

## 1 Introduction

Modern sensitivity of DNA analysis [1,2] allows scientists to obtain interpretable^1^ profiles from relatively small quantities of DNA from a wide variety of items handled or worn such as firearms, spent shell casings, clothing, tools, containers, phones, and steering wheels [[3], [4], [5], [6]]. While these modern testing methods, combined with mixture deconvolution software, are extremely efficient to obtain DNA profiles and assist an investigation aimed at generating leads by searching DNA profiles in available databases, they do not help assess what the DNA results mean within the wider case context once there is a person of interest and multiple mechanisms of DNA transfer may exist [7,8]. These questions were less prominent when the quantity of DNA required to obtain a DNA profile was large, because it was recovered from a stain that was visible and from a well-presumed body fluid. The questions of the mechanisms (or activities) that led to the visible stain or presumed body fluid were often, whether for good or bad, left to advocacy and the common sense of the factfinder. More sensitive methods allow scientists to obtain a DNA profile from a few cells without the capacity to observe a stain or be guided robustly as to the nature of the body fluid involved. In these situations, the relative weighing of the transfer mechanisms is often more difficult—to the point that today the debate evolves from the source of the DNA to questions about the mechanisms or alleged activities whereby the biological traces were transferred—signaling the scientist that they may need to re-evaluate their findings considering activity level propositions. Such an evaluation requires additional expertise and relevant case information. Although questions concerning the source of DNA have been extensively researched and are routinely addressed through laboratory training, the interpretation of DNA findings in the context of alleged activities remains less understood by laboratories and DNA scientists, despite the availability of a substantial body of published research.

When the mechanisms of transfer (including persistence) are not an issue in the case, such as when there are large volumes of blood that appear to relate directly to the alleged activity, there should be little to no risk that the decision-maker will misinterpret the DNA results in the context of the contested activities in the case [9]. If the association between the recovered DNA profile and the action(s) under investigation is undisputed and well supported by the other investigative information, then the association between the DNA profile, the person of interest, and the alleged activity build a coherent narrative the court can easily integrate into its decision making. However, when transfer is an issue, this begs a new set of questions for DNA scientists and legal practitioners including:

• How much other information in a case is needed to avoid a misunderstanding of the DNA results during an investigation or court proceeding?
• What is the nature of the expertise and knowledge required by DNA scientists to help decision makers in these cases—and how and when should such guidance be provided?

Recent National Institute of Standards and Technology (NIST) reports have renewed attention to these matters by dedicating chapters to discussions about the current state of evaluations given activities [10,11]. In [10] reiterated that DNA can be transferred and this can occur multiple times, that the value of the DNA results depends on the questions asked within the context of the case, but remained vague as to the nature of the expected testimony regarding these mechanisms. Whereas in [11], the DNA scientist was encouraged to stay in their lane of expertise and training, provide the limitations of DNA comparison values with respect to activities, and to avoid direct comment on the probability or possibility of a transfer mechanism in a specific case. Soon after, the Scientific Working group for DNA Analysis Methods (SWGDAM) weighed in—formal evaluations^2^ given activity level propositions are not suitable for use in the U.S. criminal justice system—citing scientific, legal, and practical considerations [12]. The above reports demonstrate an active and unsettled discussion on the path forward for assessing DNA results considering transfer, through a report or during testimony, in the U.S. These contrasts, and at times tensions, will be reviewed in section 4.

However, the concern over how to interpret and communicate the meaning of observed DNA results—considering the impact of transfer and persistence—is not new and we briefly refer to some court decision in England and Wales where these issues were first raised and considered by the highest court of the land [13]. In 2009, the *R v Reed and Reed*^3^ case [14] involved a relatively small amount of DNA where a reporting officer was allowed to assess possible transfer mechanisms based on her experience. There was notable commentary and concern that pointed to the sufficiency of data and the potential over-reliance on personal experience [15,16]. Shortly thereafter in 2010, *R v Weller*^4^ [17] countered on appeal with a challenge that the expert's knowledge base regarding transfer and persistence was inadequate; however, the Court of Appeals upheld the opinion on similar grounds to R v *Reed and Reed* where it was accepted that a DNA scientist may refer to their training and experience to assess the matter*.* Again, commentary followed challenging the reliance on personal opinion over peer-reviewed publication as a basis for opinions related to the meaning of DNA findings considering transfer and persistence [18]. The situation in other countries and the U.S. will be briefly addressed in section 4. A detailed analysis of recent court rulings is beyond the scope of this paper as it requires its own analysis that we aim to offer in a second contribution.

Many DNA laboratories do not have the expertise, access, or time to assess the empirical data to support the interpretation and reporting of the value of the DNA results in light of activity level propositions as they do for DNA comparisons given sub-source level propositions (see Table 2 for a description of the propositions). One reason is that assessment considering the sources of DNA is more developed with recognized databases and tested probabilistic methods, whereas the assessments of expectations regarding transfer and persistence appear to rely more heavily on the known case circumstances and on the DNA scientists’ informed judgments. This is not to suggest that there is no subjectivity with DNA comparisons, but the practice of evaluations given activity level propositions is not as well-defined or understood and is rarely reported in advance. Therefore, there are a wide variety of approaches undertaken when dealing with these questions during testimony.

It is common for DNA experts to encounter questions about how, when, or why the DNA was deposited or not [19,20]. A U.S. survey of 54 participants responses indicated that 53% of respondents would provide general qualitative statements that often transposed the conditional, for example stating that one scenario is more probable than another, while 45% indicated they would abstain from answering and may instead comment on the complex variables associated with DNA transfer [19]. This is far from ideal and has set the stage for a complicated transitional period for DNA scientists. Even if the scientist could anticipate such questions and prepare in advance of trial—what are the options available to the scientist to help the factfinder understand the DNA results in context and what guidance exists regarding how and when they should go about this challenging task? This interpretation and opinion “gray-area” is further complicated by research which has shown that laypeople tend to view forensic expert evidence as inherently trustworthy and reliable [21,22]. Moreover, laypersons may not be able to notice when an expert strays from their area of expertise especially if they are highly persuasive [23,24]. Meakin and colleagues [25] critically consider the risks of asking DNA scientists during testimony to put their results in the context of alleged activities when their submitted report assessed the DNA results only in the context of potential sources.

Specialized knowledge and expertise about DNA transfer, persistence, prevalence, recovery (TPPR) and how these factors relate to the case circumstances are required to help address such “DNA transfer” questions (*see* Appendix A. Supplementary data: Supplemental Table B. for a summary of key citations related to a DNA interpretation framework, Supplemental Table C. for an index of standards, guidelines, and other documents related to evaluative reporting, and Supplemental Table A. for a summary of DNA-TPPR and background DNA considerations). There is an absence of specialized education or formalized training programs beyond a basic understanding of DNA-TPPR in most U.S. forensic laboratories and therefore, there is not a formalized interpretation and reporting process in operating procedures. Unfortunately, the scientists are facing questions on DNA transfer articulated in real time during testimony and there are numerous human factors to consider when attempting to provide robust testimony under these circumstances [11]. The DNA scientist's struggle to communicate effectively when presented with potential explanations [26] for the DNA results can be exacerbated by the initial presentation of quantitative evidence given sub-source level propositions that only help the factfinder with the question of whether a given individual is a source of DNA or not. In these circumstances, the risk is that there is a translation (e.g., carry-over) of the value of the DNA source comparison to questions regarding the transfer mechanisms that led to the observed DNA results; these issues have been described and discussed by many authors [11,25,27,28].

Recent advances in DNA technology from probabilistic genotyping, massively parallel sequencing, to whole genome sequencing represent the changing landscape for helping to address the “who” questions, but none of these solutions help scientists with the interpretation of their biological results given activity level propositions (the “how,” “when,” or “why” questions). DNA-TPPR and background cannot be ignored in the context of testimony because these questions are systematically raised in court; however, there is clearly a lag in implementation and a lack of consensus about how and when these evaluations should be performed [29,30].

The following sections describe the current landscape for assessing DNA results when DNA transfer mechanisms are an important consideration, whether the assessment is formally interpreted and reported in advance of trial or if opinions are offered during testimony for the first time. Section 2 revisits whether the duty of the scientist is more than just helping with “who” questions, reminds of the principles of evidence interpretation [[31], [32], [33]], the importance of communicating limitations (i.e., what the findings do not mean), and closes with a discussion about how professional ethics and codes of professional responsibility require that opinions should be impartial, objective, and supported by sufficient knowledge, data, and expertise. Once one agrees that it is the scientist's duty to assist the factfinder, the problems can be boiled down to *how* (e.g., methods, data) and *when* (i.e., formally in advance and in writing or informally during testimony) the scientist should provide input. Section 3 addresses the issues confronting the scientist about how to assess their results considering DNA transfer from selection of the method and which sources of knowledge to incorporate to the complexities of communicating either the qualitative or quantitative value of the findings. Section 4 asks the question of whether these opinions should be reported or not from a best practices standpoint and provides a summary of laboratories that are formally reporting their DNA findings given alleged activities. This section considers human factors as they relate to expert testimony, the challenges of providing an opinion on the stand for the first time when DNA transfer mechanisms are an important consideration, and closes with the authors' perception of often-encountered testimony practices. Section 5 aims at supplementing recent work [34] regarding hurdles to implementation of formal methods for evaluations given activities from a U.S. perspective by discussing casework priorities, general lack of research culture, and challenges related to the legal community including literacy and perceived challenges with assigning defense propositions. The paper concludes with recommendations regarding where the U.S. community of scientists and legal practitioners should progress with these issues.

## 2 More on the duty of the forensic DNA scientist

### 2.1 Should forensic DNA scientists help with more than just questions of sources?

There does not appear to be significant debate regarding the duty of the DNA scientist to provide assistance to the decision-maker^5^ when the questions relate to whose DNA may be present; however, it is not as clear once the questions evolve to how or when the DNA may have been deposited or not.

In their assessment of recurrent concerns, Biedermann and colleagues [35] discuss the perception that the DNA scientist infringes on the duty of the court when evaluating their findings given alleged activities^6^ which appears to be primarily rooted in the problem of the transposed conditional. That is, the duty of the scientist to provide value to their results considering the propositions rather than a comment directly about the probability of the proposition itself [[31], [32], [33],^36^] and that assessing the truth of the proposition is clearly a task for the court to consider along with all the available information [35] (this is discussed more in section 4.2.1 *Amiable and mechanism-focused responses****)*.** Biedermann and colleagues reiterate that the “transposed conditional” distinction is subtle, but important—so much so that it may require the scientist to be clear about what their opinion does *not* mean [35]. In addition, the scientist will need more case information to adequately analyze and assess the findings in the context of activity level propositions which has contributed to the perception of the scientist as a general detective rather than remaining a detached scientist [37]. There is no denying that the questions are different when there is not yet a person of interest; however, it can be challenging to clearly separate between the “investigative” and “evaluative” stages of the case [38]. There are legal arguments that refer to the formulation of the alternative proposition which apply to both source and activity level issues (this is also discussed in section 5.3.2 *Can we reasonably state the defense proposition?*) and require the scientist to consider disputed facts that may not (yet) be in evidence [39] as well long-standing opposition regarding the scarcity of casework-specific DNA transfer and persistence data that should be used to inform judgment [37]. A legal scholar, Robertson, and colleagues discussed whether the scientist usurps the role of the jury and concluded that the scientist should provide their knowledge and expertise in the case by considering the activities that may have caused the trace to be transferred [40].

Recent practitioner surveys in the United States, Europe, Australia, and Canada demonstrated that there is also apprehension among DNA scientists about whether they believe it is their role to speak about the results given activities due to, in part, a lack of knowledge and comfort relating to specialized training, research, and legal acceptance [19,20]. Contrast this with publications by scientific and regulatory bodies (e.g., European Network of Forensic Science Institutes (ENFSI), Forensic Science Regulator (FSR), DNA commission of the International Society of Forensic Genetics (ISFG), ISO 21043) that discuss the need to assess biological results given activity level propositions when knowledge of transfer, persistence, and background is crucial to properly understanding the value of the results [9,27,41].

Given the varied perspectives about the scientist's obligations, will they necessarily know when it is appropriate to help address activity level propositions with their DNA results? There are some circumstances identified in the appendix of the ENFSI Best Practice Manual (BPM) that could help refine and better explain that activity level propositions are the most meaningful “when there are possible legitimate reasons for the presence of DNA from the POI^7^ (e.g., if the persons know each other or if the person has been on the premises for legitimate reasons) or when one can help assessing the absence of DNA or by considering the possibility of contamination” [42]. The examples provided in the ENFSI BPM represent potential danger zones for over-reliance on DNA results in isolation and that they should be considered in the context of the case.

The available guidance makes the most sense when considered alongside previous examples where the DNA results were misunderstood in the context of the case both by investigators and the court system to realize that an individual with the requisite expert knowledge is needed to provide assistance. There are examples of relatively well-known cases such as Adam Scott, David Butler, and Amanda Knox [[43], [44], [45]] which demonstrate situations where scientific expertise related to transfer was crucial to understand the meaning of the DNA results in the case. The U.S. cases of Lukis Anderson in 2012 [46] and Terrell Gills [47] in2015^8^ illustrate that non-criminal actions can lead to the recovery of innocently-transferred DNA, highlighting the importance of assessing the alignment of all case information as well as the risks of over-relying on DNA comparison results. A notorious example resulted in the wrongful conviction of Farah Jama in Australia where the foreign DNA profile obtained from an intimate swab aligning with Jama's profile was given undeserved weight in his rape conviction as it was the only piece of incriminating evidence that was presented [45]. Further investigation showed that contamination of the intimate swab in the examination room at the hospital led to the presence of Mr. Jama's DNA for reasons unconnected to the alleged sexual assault [48]. Judge Vincent identified the need for DNA scientist to consider their findings in the context of the alleged activities and not only with regards to the potential sources [48].

### 2.2 Communicating the limitations of evaluations of DNA comparisons

Scientists also have a role in effective communication within the wider criminal justice system—that their duty is not only to perform and report findings, but to explain what those finding do not mean, hence disclosing limitations with appropriate caveats disclosed in the report. The Expert Working Group (EWG) on Human Factors in DNA Interpretation echoed previous body's positions [9,27,49] about the importance of caveats for evaluations of DNA results given (sub)-source propositions and provided a reference Appendix 5.1 [11]. The Organization of Scientific Area Committee's Human Forensic Biology subcommittee published a technical guidance document with examples for reporting caveat language for different aspects of the DNA process [50].

Caveats ought to clarify the purpose and limitations of a report that *only* considers DNA source-seeking comparisons and the (in)significance of these results in isolation when the issues concern alleged activities. Moreover, when considering DNA transfer mechanisms, a separate evaluation of the findings is required along with specialized knowledge, training, and competency [6,11,25,51]. Examples of such caveats have also been discussed in the relevant literature [9,11,27,42,49,50].

### 2.3 Ethics, impartiality, and integrity

Most forensic associations and laboratories have a code of conduct notably to meet ISO 17025:2017 [52] that should address ethics, confidentiality, impartiality, and other content aimed at ensuring proper behavior [53]. There are several entities that have guidance and standards specific to ethical behavior in forensic science, for example from the National Commission on Forensic Science, Forensic Science Regulator, ANSI National Accreditation Board, European Network of Forensic Science Institutes, and U.S. Department of Justice [[54], [55], [56], [57], [58]]. These documents contain common themes about performing examinations, reporting, and testimonies that are impartial, objective, thorough, and warn against partisan testimony or testimony outside one's area of expertise. Moreover, these documents require the scientists to offer interpretations and opinions during testimony that are supported by sufficient data and are derived from the use of appropriate scientific principles. Texas law requires the Commission to maintain a Code of Professional Responsibility^9^ (“Code”) for forensic analysts^10^ setting the ethical standard for all licensees with the intent of ensuring that scientists and their management maintain “integrity and respect for the scientific process and encouraging transparency in forensic analysis^11^”. What sets this Code apart is that violations of the clauses, if deemed sufficiently egregious, could subject a forensic analyst to disciplinary action up to and including the loss of licensure.^12^

Justice Stephen Hall reviewed an Australian case, *Wood v The Queen*, in which a scientist for the prosecution succumb to significant issues of adversarial allegiance, a loss of independence and credibility, as well as being a victim of confirmation bias [59]. The absolute loss of objectivity was not the goal of the scientist and Justice Hall reminds the reader that this can happen all forensic scientists and adds on page 10:

“Experts have, therefore, a particularly privileged position. That privilege assumes that in giving their evidence, experts will base their opinion only on the facts and the logical conclusions that can be drawn as a matter of the application of scientific principle to those facts.” [59].

Tottenham reiterates that the primary duty of the expert witness is to *assist* the decision maker because they bring specialized or technical knowledge that the court does not have; however, this should not empower experts to give their opinion *carte blanche* [60]. Moreover, experts should have access to the necessary factual information, attempt to figure out background (uncontested) information from other disputed facts, and be even-handed and as objective as possible in applying their knowledge to the relevant facts of the case [60].

In summary, even if one acknowledges that it is the duty of the scientist to understand and communicate what the DNA results (or their absence) mean while considering transfer and persistence, the challenge lies in the ability to operationalize this approach. The current knowledge and consensus gaps appear to revolve around 1) how to interpret the results using which methods and data and 2) when this guidance should be given—in advance of trial or upon solicitation during testimony.

## 3 How to interpret

### 3.1 What is the question or issue?

Before the DNA scientist proceeds with DNA testing, they should understand the issue and what the request is asking for. They can then form an examination strategy whether the questions relate to whose DNA (source), how or when an individual's DNA could have been deposited (activity), or both. As Berger puts it in his recent publication about ISO 21043, interpretation in forensic science is about applying science to help answer questions [61]. These questions often need to be elicited by the forensic scientist. The approach has been previously formalized as the case assessment and interpretation (CAI) model, where based on the issue of the case as identified and the items collected, the scientist develops an examination strategy that can help answer case-related questions [33,38,[62], [63], [64]]. A key aspect to consider here is whether the forensic task should be investigative (i.e., provide leads in the early stages of the case) or evaluative (i.e., when there is a person of interest who may face trial and the findings need to be assessed) and whether the scientist can help with any of these tasks given the information as understood [38,49]. Table 1. Illustrates some key differences between the investigative and evaluative phases of a case; however, note that it is not always trivial to distinguish between them.

**Table 1:** Differences between investigative and evaluative phases.

Investigative phase	Evaluative phase
Tends to be crime-focused.	Tends to be suspect-focused.
Tends to be towards the beginning of the criminal justice process.	Tends to be towards the end of the criminal justice process.
Helps with investigative-process decisions.	Helps the criminal justice system take decisions.
Types of questions: What happened? What is this? Who could be involved?	Type of questions: Is Mr Smith the source of the DNA or is it someone else? Did Mr Smith drive the car or was he a passenger?
Suggests explanations for the observations and gives guidance.	Evaluates the observations (i.e., results) given at least two mutually exclusive propositions.
Open-ended process.	More formalized and structured process.
Explanations need to encompass all realistic possibilities to avoid bias and avoid potentially misleading the inquiry.	Assigned probabilities should be calibrated; rates of misleading opinions should be low and documented.

Table compares how the investigative and evaluative phase of a case impacts similar elements. Adapted with permission from [49].

**Table 2:** Evaluating biological findings given the hierarchy of propositions.

Level of hierarchy	Pairs of propositions	Results/Observations used	Factors considered	Assumption(s)
All		• DNA profile(s)		• No scene or lab contamination • No error
Sub-source	• DNA is from POI • DNA is from an unknown, unrelated individual	• The value of the comparison (LR)	• Occurrence of genotypes in the relevant population • Mixture interpretation factors	• No alternative routes for POI's DNA to transfer from provided case information: identity is the issue
Source	• Blood is from POI • Blood is from an unknown, unrelated person	• The value of the comparison (LR) • Appearance, size of stain • Screening tests	• (Factors considered given sub-source) + • Screening test false positive or negative	• No need to consider transfer or persistence due to size or quantity of material • The nature of the material is not disputed
Activity	• The POI and complainant had penile-vaginal intercourse • The POI and the complainant only danced together	• Screening tests • Absence of DNA • Relative quantity/quality of DNA (aligning with POI) • Results from multiple tested items	• (Factors considered given source) + • TPPR • Background DNA • Contamination	• The mechanics of the alleged transfers represent the case • DNA accumulates given the activities • No conditions that would impact shedder status

The hierarchy of propositions is a construct used to organize forensic issues and is presented here alongside example pairs of propositions where the contested issue is underlined. Adjacent to the propositions are the results/observations, factors, and assumptions that may need to be considered. The table is not an all-inclusive list and additional assumptions may need to be considered within the evaluation. There is little utility with source level propositions and they should only be considered if there is no dispute about the material or need to consider transfer and persistence, if there is, then the results/observations should be evaluated given activity level propositions. Table was inspired by [10,11,42,65].

Once a person of interest is identified, the laboratory analysis often shifts towards an evaluation that considers the case-relevant information and propositions which, ideally, reflect the contested issues in the case [26,49,62,66]. The principles of interpretation dictate how the scientist should assess and communicate the value of their findings considering mutually exclusive propositions whether directed to questions of sources or activities [31,33]. Table 2. Provides example pairs of propositions for sub-source, source, and activity levels in relation to the results or observations, factors, and assumptions that may need to be drawn.

As the case moves closer towards a decision (e.g., to charge, plea, render trial judgment), a current challenge is that the DNA sources questions that were previously considered may no longer be relevant if there is 1) a need to consider transfer, persistence, or background DNA (unknown DNA contributors for unknown reasons can be very important with mixtures of DNA) due to additional case information or 2) the questions evolve to the disputed activities that led to the deposition of the DNA [7]. For additional publications that discuss the DNA interpretation framework, refer to Appendix A. Supplementary data: Supplemental Tables B and C.

### 3.2 The methods

There is scientific consensus regarding the general methodological approach used to probabilistically evaluate the findings by considering at least one pair of propositions that reflect the scientist's understanding of alleged activities (refer to Appendix A. Supplementary data: Supplemental Table C. for an index of standards, guidelines, and other documents). There are also several resources that contain templates, realistic casework scenarios, and evaluations of results in prior cases [[67], [68], [69], [70], [71], [72], [73], [74], [75], [76], [77], [78], [79], [80], [81]] (refer to Appendix A. Supplementary data: Supplemental Table D. for a list of key publications). A textbook by Duncan Taylor and Bas Kokshoorn [82] was released in 2023 with content covering DNA-TPPR of biological traces, how to structure case information, basic mechanisms of evaluation (i.e., hand derivation of LRs), fundamentals of Bayesian Networks (BNs), and advanced mechanisms of evaluations [82]. Moreover, an entire chapter is dedicated to sensitivity testing which helps to ensure the robustness of the evaluation by seeing how adjustments to specific factors may impact the LR [82]. This text supplements an increasingly large volume of research publications exploring DNA-TPPR factors to include the NIST Mixture Foundation Review (Chapter 5) and the 2023-2025 Interpol review of Forensic Biology and DNA [6,10,[83], [84], [85], [86], [87], [88]].

This is not to dismiss that there are many case-specific assumptions (e.g., whether to consider contamination) and choices related to how the data may be assessed and modeled. For example, will the scientist decide to consider the presence or absence of DNA, information about mixture contributions, or DNA amounts, each of which could lead to different likelihood ratio values [30,82]. Additional interlaboratory studies are necessary to continue to explore the extent of variability related to these choices.

More significant gaps in general agreement appear to focus on the sources and sufficiency of the knowledge referred to during an evaluation, the requisite expertise required to inform these assessments, and the communicative challenges surrounding the evaluations.

### 3.3 Source of knowledge

Emerging from the review of the current state of guidelines and published discussion is the preference to use data obtained from controlled ground truth experiments whether from empirically-derived publication or case-specific experiments, although this option is likely the least feasible [9,13,27,82,84,89]. The ENFSI guidelines [9] glossary defines data as the “technical and empirical knowledge associated with a given trace type.” The FSR guidance for interpretation and communication released in 2026 [90], with guidance related directly to testimony, aligns with previous documents that the assignment of probabilities should be informed by structured, published data where such data exist. In the absence of published data, there are allowances in available guidelines to use personal casework experience as a source of data [9,41]; for example, in the recent FSR guidance [90], indicates that when there is not “relevant and robust experimental data” the expert may have “sufficient personal, professional experience and knowledge” but, and this is critical, expertise such as this “should be calibrated regularly through collaboration and participation in activities such as proficiency tests.” Martire and colleagues [91] assessed the performance of human expert judgment in the handwriting discipline and concluded that “a cautious approach should be taken before endorsing the use of experience-based likelihood ratios for forensic purposes in the future.” Other DNA-specific documents, including ISFG part II guidelines, offer ample warning against strictly relying on personal experience or intuition [13,25,27] because even DNA scientists can have misconceptions (ideas which might not be supported by available research) about how DNA results relate to their expectations of DNA transfer and persistence [25,51].

When assessing the current landscape, expert knowledge based solely on training and experience appears to be the least desirable of data sources. Expert knowledge undeniably relies on training from ground truth experimentation, but it is the articulation of the reasoning and justification for the knowledge or data considered that is crucial to the evaluative process—whether it pertains to the suitability of an evaluation, how the data is used, or the extent of any adjustments. Decisions related to how the chosen data reflect the case circumstances and any adjustments are supported by specialized knowledge and does not diminish a data driven approach [82,92].

### 3.4 Quantitative or qualitative assessment

Taken together with the previously discussed method, the scientist will still have a choice of how to report and communicate the strength of their results. Available standards and guidelines have created an ambiguous situation surrounding whether the expert should communicate in a quantitative (i.e., numbers) or qualitative (i.e., words) format. The ENFSI guideline requires the scientist to assign a numerical probability rather than an undefined qualifier and note that if a LR cannot be assigned then no assessment can be made [9]. The ISFG part II guideline makes also clear that “value” means that a number is assigned which may be followed by a verbal qualifier [27].

Other documents, such as the UK Forensic Science Regulator (FSR) codes of practice for evaluative opinion [41] and NIFS guide to evaluative reporting [93], are less restrictive with respect to numerical assignment and allow experts to assess value qualitatively in appropriate circumstances. The FSR guidance document released in 2026 generally permits expert opinion where it is relevant to the matter at issue, the expert is competent to provide it, and the opinion is sufficiently reliable to be admissible [90]. Regarding opinions at the activity level, the guidance states that experts should use appropriate and precise language and clearly communicate any limitations affecting the interpretation [90]. The FSR guidance further describes a tiered approach to communicating evidential value: (1) a numerical likelihood ratio (LR) is preferred where robust and structured data are available; (2) a range or order of magnitude may be communicated where the available data are limited; and (3) where no inspectable data are available and the assessment relies, for example, on personal experience, a “narrative” LR or verbal expression may be used, accompanied by explicit disclosure of the associated limitations, including whether the underlying knowledge has been calibrated and whether a broad range of expert opinion is likely [90].

A similar emphasis on the basis, expression, and limitations of forensic opinion is reflected in the ISO 21043 Forensic Sciences standard series. Parts 3 (*Analysis*), 4 (*Interpretation*), and 5 (*Reporting*) were published in 2025 [[94], [95], [96]]. Part 4 [95] describes different types of forensic opinions including general, likelihood ratios, and categorical opinions, and addresses how they may be expressed, including in generally, qualitatively, or quantitatively. Part 5 [96] addresses the reporting and testimony of opinions, with particular attention to the basis for the opinion, its limitations, and the review of opinions presenting greater risk, including those based directly on human judgment.

Berger [61] appears to question a strict distinction between qualitative and quantitative opinions, noting that both ultimately involve an assignment by the expert. A quantitative opinion may be generated numerically through a model, whereas a qualitative opinion may rely on a predefined scale associated with ranges of numerical values. Regardless, ISO 21043 uses ‘opinion’ for both qualitative expert opinion and those based on a statistical model and the standard series appears to be moving forensic science towards more reliable expert opinions by emphasizing ‘what’ should be accomplished rather than ‘how’ [61].

The “words versus numbers” topic was examined by the Texas Forensic Science Commission (TFSC) which related to the first *known* case in Texas v. Armstrong^13^ where an expert attempted to offer testimony following an evaluation of DNA results considering alleged activities in advance of trial [97]. The investigation and subsequent report released in July 2024 explored many aspects of what interpretation, reporting, and testimony might look like in the United States [97]. A key takeaway was whether numerical likelihood ratios should be required as opposed to a verbal qualifier or other qualitative terms to describe the strength of DNA results in the context of alleged activities. To our knowledge, this was the first time this issue had been formally raised in the United States. After the TFSC report, Buckleton and colleagues [98] attempted to reconcile the available recommendations regarding whether likelihood ratios must be solely expressed numerically. They wrote that the presence of a numerical LR alone does not guarantee methodological rigor and propose that the community should decide whether numerical reporting should be mandatory [98]. Notably, however, the same comparison paper includes consensus recommendation 15^14^ which specifies that the LR shall be reported in numerical form [98].

Two case reports were published in 2025 where a formal approach to an evaluation of the DNA findings using a BN—resulting in numerical LRs— on two different U.S. cases that had been to trial. One case report stated that the previous trial testimony about the meaning of the DNA results in *Texas v Armstrong*, in qualitative terms such as they are “more likely” or “much more likely” was justifiable due to the LR value of about 1300 in favor of H_1_ [81]. A subsequent case report assessed DNA results where there was a reported dispute regarding how DNA transfer was discussed in light of the alleged activities in *Massachusetts v Richardson* due to the LR value of about 800 [80]. A couple key learning points from these reports are 1) given proper time and consideration, evaluations of results given activities resulting in numerical assignment of value can be performed and 2) it is reasonable to specify in a transparent manner the basis of the opinion including the assumptions and factors considered during an evaluation.

Another consideration is that the magnitudes of LR values assigned to results given activities will often be several orders of magnitude lower than LRs given sub-source level propositions when supporting the prosecution's view [27]. In other words, these LR values may not often fall in the “Strong Support” (10,000 - <1,000,000) and “Very Strong Support” (≥1,000,000) categories [99].^15^ As discussed by the EWG on Human Factors in DNA Interpretation, there are many nuances to consider with the implementation of a verbal scale to include how different words are interpreted by different individuals, but there should not be different scales for different disciplines or methods [11]. The FSR guidance encourages a “core verbal scale with discipline-specific interpretation and a strong emphasis on the primacy of the LR” to support continuity and explicitly prohibits “back-transformation” where a numerical LR is inferred from a verbal term [90].

Logical reasoning does not strictly require the assignment of a number to express evidentiary strength [100,101] and this is reflected in the flexibility of the available standards and guidelines which allow for expression of qualitative opinion. That being said, where sufficient case information and structured data exist, there is a consistent preference for reporting numerical values [9,27,90,93] and Taylor & Kokshoorn's textbook [82] as well as the continued efforts to collect, standardize, and share DNA-TPPR research and establish a repository of Bayesian networks [25,30,87,89,102,103]. These aspects are discussed further in Section 5.2.

### 3.5 Comprehensibility and communication

When examining what successful communication of scientific findings might look like, it is important to look at the comprehension of the end-user and if there are differences between how results (or conclusions) are presented (see Ref. [104] for a review and gap analysis). Lay comprehension of value statements is a challenging area of study because there are many aspects to consider regarding consistency, framing, presentation format, and whether lay individuals think in line with Bayesian logic regarding the updating of belief accounting for the reported statistical value [105]. Bali and Martire found no significant impact of conclusion format (i.e., RMP, LR, verbal term,^16^ or categorical) on lay assessments which ultimately reinforces the recommendation to use scientifically sound conclusion format such as likelihood ratios for presenting evidence within expert reports since there is no hindrance to understanding compared to simpler categorical formats [106]. These results do appear to conflict with previous other studies which have indicated format-effects on trial-related decisions [[107], [108], [109]]. A key difference of the Bali and Martire study is that participants were given an *entire* expert report as opposed to snippets—their findings also suggest the empirical importance of other features of expert reports in shaping how laypeople evaluate forensic expert evidence [106].

A 2025 review article by Morrison, Martire, and colleagues [110] examined research through the lens of CASOC^17^ indicators [105,111,112] to try to determine the best way to present likelihood ratios to maximize their understandability for legal decision-makers. The authors used refined definitions of sensitivity,^18^ orthodoxy,^19^ and coherence^20^ to compare different formats to express a likelihood ratio (LR): numerical LR, numerical random-match probabilities, and verbal strength of support statements [110]. As a result of their extensive review, Morrison and colleagues [110] made a recommendation, supplemented by the methodological issues observed and their own insights, that based on theoretical and logical grounds,^21^ numerical LRs should be used and future research should focus on their presentation especially during testimony.

Effective communication of the strength of evidence—whether qualitative or quantitative—continues to be one of the most challenging aspects of being a forensic scientist who must interact with a variety of end-users. Expressing a numerical or non-numerical likelihood ratio was discussed over 15 years ago as *one* aspect of the Court of Appeal of England and Wales' decision in the case of *R v T.*^22^ The decision ignited several commentaries (for example [100,101,[113], [114], [115]]) about the Bayesian approach to evidence evaluation. Berger and colleagues discuss that although it is not necessary to have a purely mathematical method from databases to assign a likelihood ratio in a logically supportable way—it is quite problematic to “cite years of service, numbers of previous cases and expect the Court to accept it *ipse dixit”* [100]. Similarly, Morrison wrote that there is a difference between the degree of subjectivity inherent in building a statistical model to calculate a numeric LR versus the assignment of a LR “based entirely on the experience and implicit knowledge of a forensic expert” and when discussing verbal scales “[w]henever possible, strengths of evidence should be presented as numeric likelihood ratios calculated on the basis of databases, objective measurements, and statistical models …” [113]. This sentiment is echoed by Martire and colleagues who discuss the extent to which personal probability assignments are helpful—rather than misleading statements of faith—to the court considering their role in assessing the rigor of such statements [116]. Morrison also wrote that a primary concern of the court appeared to be the absence of disclosure for the underlying calculations in a transparent report to support the non-numerical opinion given by the footwear expert during the *R v T* trial [113]. Many authors have since discussed the importance of transparency and disclosure of the basis for the scientist's opinion [11,25,117,118] which leads us to a discussion about whether an evaluation of findings given activities should be reported or not.

## 4 Reporting DNA results considering alleged activities

### 4.1 To report or not

In the United States, it is standard practice to provide a report that evaluates the DNA comparison results when it comes to their sources [52,119]; however, this is not the case when it relates to activity level propositions. This is evident in SWGDAM's recently released position statement that there were scientific, practical, and legal challenges to “activity level reporting” in its present form and that it “is not suitable for use in the U.S. criminal justice system” [12]. The statement closes with the sentiment that analysts will continue to face TPPR questions, presumably SWGDAM means during testimony, and that they will explore alternatives to the formal ALR approach [12]. Refer to Section 5. for more discussion as it relates to the challenges noted in the position statement.

The Human Factors EWG recommended that scientists avoid conducting new evaluations during testimony because the witness-stand setting may not provide the time, information, or working conditions necessary to perform a thorough interpretation and subject it to technical review [11]. Applying this recommendation in practice, however, can be challenging because there may not be broad agreement about which statements or opinions constitute a new evaluation. Some situations are relatively clear, such as when an expert is asked to interpret a comparison that was not previously performed or to calculate statistics in the witness box. Other situations are less readily defined, particularly when questions concern DNA transfer where questions may range from general inquiries about the expert's knowledge to more complex, case-specific assessments requiring consideration of the relevant literature and an assignment of relative value given the proposed mechanisms. In the absence of clearer guidance, experts may exercise considerable discretion in determining the extent to which they can appropriately respond, which may result in variability in both the scope and substance of testimony.

Standards, guidelines, and several other publications point to the necessity of reporting which emphasize transparency and disclosure, requiring the expert to justify the basis of their evaluation, the information considered, assumptions drawn, and the source of their knowledge used to assign probabilities [9,11,27,33,41,42,49,82,93,98,117,118,120]. The International Organization for Standardization (ISO) published ISO 21043 *Forensic* Sciences series of standards^23^ (Parts 3, 4, and 5 [[94], [95], [96]]) in 2025. While *Part 3: Analysis* and *Part 4: Interpretation* categorize “activity” under the Reconstruction section, there is no specific mention of activity in *Part 5: Reporting* outside of adding a caveat to a report considering only questions of sources that it does not help address the activity through which the item was transferred. *Part 5: Reporting* provides general requirements that examiners shall not report beyond their area of expertise or what can be based on the available information. It also requires that reported opinions shall conform to Part 4, to include providing the basis of the opinion and under the section dedicated to testimony, requires that “[o]bservations and opinions should be supported by either reports, case records data or documents, or all.” In their review, Stacey and colleagues discuss whether the ISO 21043 series of standards—as they relate to evaluations given activities (they use ALR to indicate activity level reporting)— are sufficiently detailed to create a quality assurance system for ALR [34].

All of the available codes of practice, guidelines, and standards have a significant shortcoming in that their adoption is voluntary. The documents also lend themselves, particularly when assessed as a group, to variable interpretations about exactly what is required and when it is required for an interpretation and resulting opinion—written in a report or spoken during testimony. Stacey and colleagues noted the confusion created when assessing available guidelines for how these evaluations should be reported [34] shortly after Buckleton and his colleagues attempted to reconcile the community's publications to form consensus recommendations [98].

An example of a case where there are different ways to interpret the available standards and guidance was illustrated in Texas where an expert attempted to offer testimony about the DNA results given activities in which the expert prepared some documentation but no formal report was provided [97]. The TFSC's aforementioned investigative report encompassed a systems-approach to assessing the nature of the issues regarding the formation of evaluative testimony—among many recommendations was a clear takeaway regarding the importance of a report to help ensure transparency about the basis for the expert's opinion [97]. The TFSC report also offered a couple of recommendations regarding testimony under these circumstances on pages 66-67:

“(3) Issue a written report containing balanced, logical, transparent and robust evaluations, being clear about their assumptions and the (statistical) model used to assign probabilities;

(7) Decline to offer ad-hoc pseudo evaluations on the stand by following the *ENFSI 2022 Best Practices Manual* on how to respond in circumstances where additional information would be needed to provide a proper evaluation.” [97].

Though best practices indicate support for reporting opinions in advance, this does not reflect the majority of practice surrounding the communication of the meaning of DNA results given alleged activities. While there are no currently known accredited U.S. laboratories authoring such reports, there are some examples of non-U.S. laboratories that have taken on a formalized approach to reporting given activities in advance of trial. Table 3 below presents the known laboratories that responded to an inquiry for data about how they provide formalized notices in advance of trial. Table 3 may not represent every laboratory that formally reports these findings and should not be interpreted as a complete representation of the current state and volume of reporting.

**Table 3:** The status of some non-U.S. laboratories regarding formal evaluations given activities.

Entity	Year Began	Current Approach	Future Direction	Requests	Reports	Testimony/Court use
University Center of Legal Medicine (Lausanne-Geneva, Switzerland) [121]	2019	Quantitative report given activities (BN or formulae)	NA	27	15	1
Netherlands Forensic Institute [122,123] *(as of 2023)*	2013	Quantitative report given activities (BN-binary)	Quantitative report given activities (BN-continuous)	74	65 (59 with conclusion)	57 Verdicts
Oslo University Hospital [124]	2010	Reports with general information and references to relevant literature	Quantitative report given activities (BN)	>90	>90	N/A
Swedish National Forensic Centre, Swedish Police Authority [125]	2018	Quantitative report given activities (BN)	NA	NA	15	5
Forensic Science South Australia [126]	2017	Quantitative report given activities (BN-continuous)	NA	20	40	1
Ontario Centre of Forensic Sciences [127]	2017	Letter of Opinion (LOP) given activities (qualitative)	Quantitative report given activities (BN)	NA	9 (LOP)	5
Victoria Police Forensic Services Department [128]	2021	Qualitative report given activities	Quantitative report given activities (BN)	>20	9	6

Content and metrics were provided directly from the laboratories and represent estimates where available. If the data was not tracked or was determined to be too difficult to compile, it was listed as not available (N/A). Participating laboratories were aware that these data would be published.

In 2023, a retrospective of the work performed since 2013 by the Netherlands Forensic Institute (NFI) was published which also included information about the Netherlands Register for Court Experts (NRGD) subfields in forensic biology to include “DNA Activity Level,” the contents of their reports, and an assessment of how their reports were used in the courts [122]. The NFI currently uses a binary method (e.g., presence or absence of DNA) in their quantitative evaluations and plans to move towards implementing tools that consider DNA quantities (e.g., continuous methods) [123]. Oslo University Hospital has been reporting general information with specific references to literature since 2010 and is currently working towards implementing quantitative reporting using Bayesian networks [124]. The Swedish National Forensic Centre began with qualitative reporting in 2018 and transitioned to quantitative reporting using Bayesian networks in 2024 where the categories for evaluation (e.g., binary: presence/absence; relative quantities: major/minor/balanced, large/small/no DNA) depend on the available data [125]. Forensic Science South Australia (FSSA) has fewer requests than reports because initially the scientists themselves were driving the request for reports as opposed to an external entity [126]. The FSSA has always reported their evaluations numerically, but transitioned over time from initially considering only presence or absence to currently considering DNA amounts in every evaluation, which was implemented in 2024 [126]. The Ontario Centre for Forensic Sciences has been issuing a letter of opinion (LOP) in advance of trial since 2017 where they provide a qualitative evaluation of the test result(s) considering two activity level propositions [127]. The laboratory updated their LOP framework to formalize the process of providing reports given activities in 2024 and have issued two LOPs since [127]. Victoria Police Forensic Services estimated the number of the requests to be at least 20; however, this number is not tracked well and may be much higher for cases where the contested issue of activities is identified at acceptance since the estimate was based on the cases completed and the case had progressed towards a judicial outcome with prosecution and defense assigned [128]. Although Forensic Science Ireland (FSI) was unable to provide specific reporting metrics, they provided general information on their processes to include their ability to rigorously pre-assess cases using the CAI model, which are peer reviewed, and to pro-actively evaluate findings given activity level propositions when it adds value to the interpretation of the evidence and there is sufficient information [129].

Although the laboratories above represent a small number of the global forensic DNA laboratories, they demonstrate that formal training, which has been in place for over 10 years at some institutions, is achievable and that there are a variety of reporting approaches to choose from. It is worth asking why it is not more common to give advanced notice of the opinions in anticipation of trial? While each lab will face their own challenges and priorities which will impact implementation of advanced methods of interpretation, the commonality between each of the above laboratories are individuals within their labs that have historically provided specialized training to other scientists for such evaluations or have received that training themselves. As transparency is key, this approach will go towards right-sizing or correctly valuating the DNA findings related to how they were assessed to include the sources of data so that these issues can be litigated in advance of trial.

### 4.2 Landscape of current testimony options regarding DNA transfer

Trial testimony is an incredibly difficult task under the best of circumstances and there are many human factors to consider when communicating during testimony. The DNA expert will need to convert complex jargon to comprehensible statements that lay individuals can better understand, find ways to reduce cognitive load such as cadence, volume, and conciseness, and cope with the stress, anticipation, and fatigue that results from testimony in an adversarial setting [11,[130], [131], [132], [133]]. These stressors combined with the presentation of incomplete information by an advocate even further complicate the expert's task when deciding how to respond to questions. This intersection between scientific communication and legal strategy is difficult to navigate.

DNA scientists also may not universally recognize that when they are asked seemingly basic “DNA transfer” questions that they may be moving into an area of testimony that is intended to help address the disputed activities [19,20] and that transfer is just one of many factors including persistence, prevalence, background, and contamination that may need to be considered.

The DNA expert finds themselves in a position where they have little control over the questions they are asked, particularly when the questions relate to how or when that DNA may have been deposited.

The DNA expert must keep in mind that their testimony carries significant weight in the criminal justice system—it is highly regarded as offering impartial and scientifically based evidence—and that when the expert is in a zone of subjectivity this is where extraneous influences can have a significant impact on their opinion [134]. The DNA scientist's expectations about the outcomes of DNA results given specific circumstances is shaped by experience; but their opinion should be more than just their best guess [87]. Experience is also shaped by exposure, and DNA scientists may have a better understanding about “typical” mechanisms rather than secondary transfer or contamination mechanisms that are perceived as rare. Experts should also not forget that they can simply respond that they “do not know what happened” or that they “do not have enough information to help address that question.” Experts may be able to provide gentle reminders to the court that the meaning of the DNA results changes relative to the questions and that regardless, they are one piece of the puzzle [[9], [10], [11],^49^].

Therefore, even though the DNA expert brings more specialized knowledge to the court than the factfinder has, the scientist must assess the risk on a case-by-case basis of providing (or not) additional guidance to the court about DNA transfer-related questions. Taylor & Kokshoorn aptly state the following about court testimony in their textbook on page 372:

“We must recognize that it is not possible to perform a formal evaluation of the case findings on the stand. Acquiring the task-relevant information, setting propositions and assigning a probability to the findings given the set of task-relevant information, while basing yourself on relevant scientific studies, are simply not something that can be done on the fly. Such an evaluation requires a thorough study of the relevant literature, assigning probabilities based on that data and performing sensitivity analyses to make sure that the evaluation (and the resulting LR) is robust. It is therefore important that all parties in a jurisdiction understand that such issues are best put to the scientist before the trial.”

There are many options, or combinations of them, for the DNA scientist to consider during expert witness testimony when asked questions about how or when the DNA was deposited (i.e., actions or timings that could have led to the observed DNA profiles). Table 4 summarizes the authors' perception of the current state of testimony *responses* when there has not been a formal evaluation of the results given activity level propositions in advance of trial. The authors are not necessarily advocating for or against the listed approaches. The responses below were organized by the authors’ perceived level of complexity and the amount of preparation that would be required before trial.

**Table 4:** Categories of testimony when asked questions about “DNA transfer”.

Response Category	Description	Why may be used
Amiable	The expert acknowledges that most proposals are possible.	Requires no preparation or application of expertise; the scientist will also appear friendly and not evasive.
Mechanism-focused	The expert directly opines on whether a certain transfer mechanism, action, or timing is more likely than another.	Requires little preparation and is based on their general understanding of literature—may rely on the intuition of the scientist.
Avoidance with limitations	The expert answers a “transfer” question with a reiteration of the limitations of DNA comparisons—the value of the DNA comparison should not be applied to questions about how or when.	The scientist stays in their lane of evaluations of the DNA comparison, repeats the limitations, and refuses to give any information about disputed activities.
General knowledge	The expert provides general and educational information to the jury about transfer and persistence.	A way to help the factfinder understand the variety of DNA-TPPR factors that must be considered without providing a case-specific opinion.
Specific knowledge	The expert conveys information from a credible source (e.g., published literature, in-house study) that relates to the case facts.	A way to give DNA-TPPR knowledge to the factfinder can use to understand the DNA results in the case.
Qualitative statements	The expert uses a descriptive term to give relative value to the results in reference to two opposing activities.	When the expert is unable to give a numerical value to the results given the disputed activities but determines that the results provide more support for one view than the other.
Numerical statements	The expert may give a range or a single numerical LR value of the DNA results in light of disputed activities.	The opinion relies on a model or BN that can be used for a variety of circumstances and DNA results, such as a look-up table.

This table summarizes the authors’ view of the potential response categories to questions about DNA transfer or alleged activities that may have led to the observed DNA results in the case which often occur for the first-time during trial. In addition to a description, each response category provides reasons why the particular responses may be used.

#### 4.2.1 Amiable and mechanism-focused responses

Amiable and mechanism-focused responses inherently transpose the conditional and violate scientific principles of evidence interpretation [31,32,36] by either agreeing a transfer mechanism is possible or that one mechanism is more likely than another. Giving an opinion about the most likely way of transfer is warned against in many guidance documents both internationally and in the U.S. [9,11,27]. Simple acknowledgements and explanations [26,135], such as “could have” or “the results are consistent with that activity” can misdirect the end-user regarding how to understand what the DNA results mean in the case whether due to an “impressive number” from a DNA comparison or the absence of results [28,32]. Moreover, explanations are merely a selection of a few (non-exhaustive) possibilities that could align with the observed results and are not evaluative in that the results could be observed under many different explanations where the scientist is unable to inform the decision-maker about how often they might see the DNA results under any one explanation [38,136].

There is available case law^24^ on the transposed conditional as it pertains to source level questions, but there has been a reluctance to restrict expert witness testimony when it comes to hypothetical activity level questions about what mechanisms could have caused the observed DNA results—after all, experts are allowed to give opinion testimony per the Federal Rules of Evidence, Rule 702 [137]. Hunt discussed the historical aspect of the role of the expert witness, the provision of explanations, and how the rules of evidence have shaped how current expert witness testimony is given on pages 45-46:

“The U.S. rules of evidence, which govern the role of expert witnesses, were developed in that context with the *explanation* of scientific issues in mind, rather than simply the *interpretation* of the evidence given those explanations. This historical point is not an endorsement of the current state of informal activity level testimony in U.S. courts. Rather, it helps explain why such testimony evolved to its present state—in concert with the common law and modern rules of evidence. However, the appropriate *scientific limits* on informal activity level testimony are a separate matter from the *legal latitude* given such testimony by U.S. evidence rules and caselaw.”^25^ [39]

Regardless of the legal latitude given, the DNA expert has control over how they respond to a question that asks them to comment directly on a scenario and they are not forced to transpose the conditional.

#### 4.2.2 Avoidance with limitations responses

Avoidance with limitations responses do not answer the question that was asked, at least with a simple yes or no. Instead, the scientist answers by reiterating the limitations of the DNA interpretation (given a comparison)—that the interpretation of the DNA results given questions of source does not help address the activities—however, there is no guarantee that the factfinder will understand this warning. The witness could also be perceived as evasive if they do not answer the questions asked by the attorneys and could be instructed by the court to do so.

The EWG on Human Factors in DNA Interpretation and TFSC have written about the reiteration of limitations during testimony [11,97] as discussed in Section 2.2.

A criticism of this approach is that, by declining to address activity-level questions, the analyst may do a disservice to the factfinder by failing to explain what the DNA results mean in the context of the case. On this topic, Hunt [138] wrote about a DNA analyst refusing to answer an activity level question that reflected the exact alleged circumstances of the case [139]. Hunt discussed that the DNA expert's credibility was undermined and that the DNA results (addressing potential sources) were given little weight, and closed with a call for the community to develop forensic guidance that considers both scientific and legal perspectives for how to address activity level questions that arise for the first-time during testimony [138]. Ideally, the expert would be given the opportunity to explain to the court the factors they would need to consider when answering activity level questions.

#### 4.2.3 General knowledge responses

General knowledge responses involve a concise education of the factfinder about DNA-TPPR factors and has been referred to as framework testimony [140,141]. This approach could help adjust jury expectations as well as dispel common misconceptions about the mere presence of a DNA profile, such as from the NIST mixture foundation report's discussion that it is possible to handle an item without transferring detectable DNA or detected DNA may be present due to indirect transfer, whether from a person or an object [10]. Of 114 respondents, 51 (∼45%) stated that they would provide general information on DNA persistence and transfer based on published data [20].

A challenge to this approach related to considerations of DNA-TPPR, often used with eyewitness testimony and psychological conditions, is that generalized information potentially lacks specificity to the case—whether legal or empirical “fit”—and could be deemed inadmissible in court [140]. Another criticism of this method is that by providing only general DNA-TPPR information, the scientist still shifts the burden of DNA interpretation considering transfer to individuals who lack specialized knowledge which could impact their ability to properly combine the DNA results with the other available information in the case.

#### 4.2.4 Specific knowledge responses

Specific knowledge responses aim to incorporate published or laboratory-generated data to help address, for example, how often one might observe a cohabitator or an unknown individual's DNA underneath their fingernails. The expert would have to prepare for this in advance of testimony and bring along the necessary supporting materials. This response is the first category from Table 4 that attempts to use the expert's knowledge about DNA-TPPR to help the factfinder understand the results *in the case at hand.* However, this approach is incomplete and imbalanced if only considering the likelihood of the results given only one point of view because an LR depends on the findings with respect to two different propositions.

#### 4.2.5 Qualitative statements

Qualitative responses (referred to as ‘descriptive assessments’ in FSR's GUI-0004 [90]) attempt to give relative support for the observations, for example by stating the results are “more likely,” when considering one activity, or transfer mechanism, versus another. Similar to specific knowledge, the expert would need to prepare in advance of trial, bring pertinent papers, and refer to supporting data as the basis for the reasoning used to support such an opinion, in addition to avoiding the transposed conditional. Anticipating questions from both prosecutors and defense attorneys represents a significant challenge, especially when the DNA expert may not know (and perhaps should not know as this is not the scientist's role) the legal strategy in the case.

The expert may also encounter challenges explaining what is meant by “more likely” when there is no numerical reference to evidential strength or a basis beyond their (un)calibrated experience to justify the statement. The context of the opinion is important and substituting a verbal expression for a probability (or ratio of probabilities) can be problematic because a non-expert should not be expected to distinguish between terms “likely” and “very likely” particularly without a scale to [136]. The use of verbal qualifier statements such as those used to supplement an LR given a DNA comparison should not be confused with this approach or used to infer a LR magnitude (back-transformation per [90]) when a numerical value has not been assigned. The FSR guidance document [90] discusses that a clear articulation of the reasoning and limitations should accompany a descriptive assessment and the “inherent uncertainty and potential for variance of opinion should be acknowledged.” This variance of opinion will exist whether the evaluation is qualitative or quantitative; however, if the opinion is based on experience or if there is not much published data available, “there is likely to be a broad range of expert opinion” [90].

Sections 3.3, 3.4, 3.5 discuss the challenges to this approach in more depth.

#### 4.2.6 Numerical statements

A numerical statement consists of a numerical expression of value assigned to the observations in respect to two competing activities. The value might be based on published papers that contain models of primary and secondary transfer where the expert could opt to give a range of LR values based on DNA quantities when considering generalized circumstances [67]. Other proposals for numerical assignment of value could be to use a sensitivity analysis [142,143] such as in Buckleton's case report [81]. A look-up table was designed to help assign a LR given the circumstances as understood and the observed DNA results as seen by another case report by Buckleton and colleagues where DNA results obtained from fingernails were evaluated relative to whether an attack was alleged, scratching occurred, and a classification of social interactions were provided [144]**.** Predictably, the expert would need to prepare in advance of trial, bring pertinent papers and data, and also understand the methods and models referred to.

The DNA scientist may face challenges about whether the case information given during testimony is adequate enough to use a look-up table. The scientist should also understand the components of the table, be prepared to explain their reasoning, and justify the underlying assumptions and probability assignments inherent to the evaluation.

### 4.3 Alignment and discretion

A consistent range of guidance and standards instructs the scientist to *avoid* commenting directly on a scenario, or comparing scenarios, during testimony [9,11,27,41,90,93,95,96,98]. Though legally allowable, these statements may give an impression of scientific validity since they are being stated by a scientific expert. However, this nuance may not be well understood by DNA scientists (or receivers of the information) when the questions—which may appear non-specific or exploratory—shift from source to activity. The key appears to be education and the extent which is required to render justifiable opinions when the issues regard how or when the DNA may have been deposited. The EWG in Human Factors considers this issue on page 175:

“The EWG considers the present situation problematic, where analysts are providing answers to activity-level questions during testimony by acknowledging that a particular mode of transfer is possible. Moreover, it would be problematic to give an opinion on the value of their results considering factors such as transfer and background, without having sufficient validated methods, training and education, competency testing, and quality system assurances available.” [11].

The need of education was also highlighted in a recommendation by the TFSC on page 66:

“(2) Obtain education and training at the level discussed by Taylor and Kokshoorn in *Forensic DNA Trace Evidence Interpretation* (2023), *before* offering services as an expert in an evaluation of biological results given activity level propositions.” [97].

The knowledge base for opinions about results given activities based solely on personal intuition or experience are equally difficult to justify and challenge as they represent an unsubstantiated opposing view or “statement of faith” [13,145,146]. Moreover, evaluations supported by generally held beliefs based on experience represents a poor substitute for empirically derived data from ground truth studies [25,27]. If the scientific expert decides to help address what their results mean when considering DNA transfer (and related factors), they will need to be forthright about what type of information and knowledge (e.g., source of data, basis and strength of opinion) would be necessary to support a transparent and robust opinion.

Alternatively, following standard warnings, the expert could explain the difficultly of the evaluative process given disputed activities which may give the factfinder the necessary context that these opinions are not simple to derive—or intuitive— and require considerable education, preparation, and effort. Either way, the scientist will find themself in a difficult position during testimony in the absence of a formalized evaluation that was performed in advance of trial.

## 5 Hurdles to implementation

Previous publications have discussed common themes related to the hurdles associated with implementing a formal evaluation of results given activity level propositions and the authors here do not intend to re-hash these concepts as they still represent hurdles that have not been overcome. The authors point to Refs. [6,25,29,51,89] include discussions on required training, harmonization of research data, and standardization of methods to include those covering training, interpretation, and reporting. Additionally, the recent review by Stacey et al. [34] considers global barriers to adoption and discusses the state of TPPR knowledge, the extent of required training for gaining expertise, and the available guidelines and standard. There is now a textbook by Taylor & Kokshoorn that provides a comprehensive foundation for evaluating trace DNA results considering activity level propositions by covering the methods for evaluation, fundamentals of Bayesian networks, sensitivity testing, reporting, and implementation which includes example procedures [82].

**Fig. 1:**
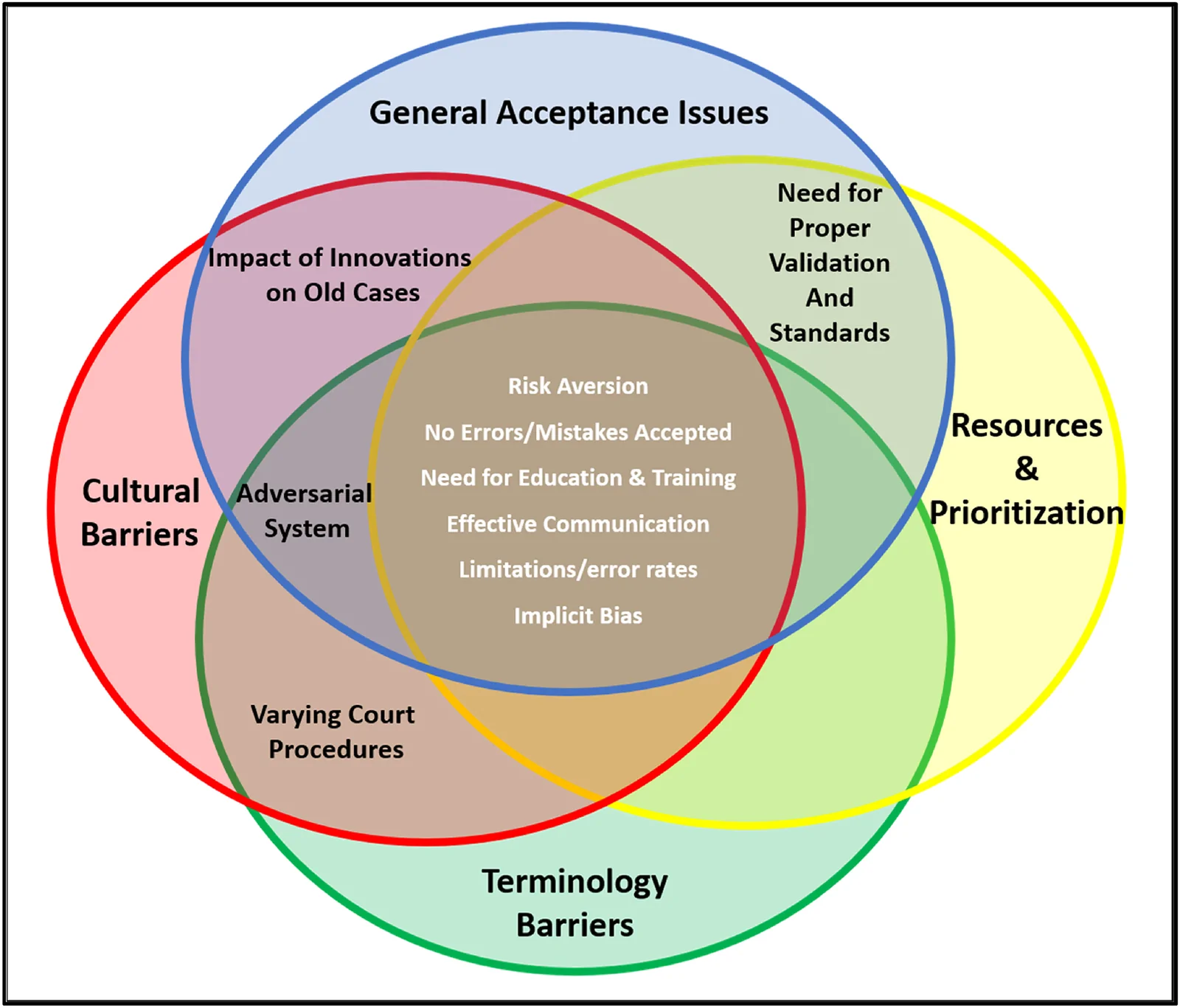
This image describes the barriers when considering the implementation of any new forensic science technology, instrumentation, or software. Figure taken from [147] with permission.

This discussion will attempt to isolate a few points of interest to the authors inspired, in part, by Fig. 1 which was first published in a NIST special report (and later re-published in the EWG on Human Factors report [11]) which discussed how the barriers for the implementation of a new technology are interconnected and cannot be separated [147]. The complexity represented in this image serves as a fitting demonstrative for all the factors to be considered for implementing evaluations of results given activities.

### 5.1 Resources and prioritization

#### 5.1.1 DNA case backlogs

DNA laboratories continue to suffer from significant backlogs, notably in the U.S., and this is despite continued federal support for publicly funded DNA laboratories. Per a Bureau of Justice Assistance fact sheet, the DNA Capacity Enhancement for Backlog Reduction (CEBR) program consistently awards over $100 billion dollars to over 100 service providers to carry out DNA testing with the specific goal of developing and entering profiles in the CODIS database.^26^ In a 2022 congressional research article [148], jurisdictions that received capacity enhancement backlog reduction (CEBR) grants from 2011 to 2017 experienced an increase in casework output (from 200,000 to 250,000); however, this was off-set by an approximately 20% increase in requests resulting in an approximate 85% increase in backlog. Further complicating this issue in some U.S. states are the existence of laws that mandate 90-day turnaround times for sexual assault kits^27^ and the extent of testing required for capital murder cases.^28^ It is in part due to these laws that the TFSC's Armstrong report [97] references the volume of felony cases and existing backlogs. Hunt [39] also mentions the resources that would be needed to implement formal evaluations of results given activity level propositions due to the sheer number of cases in the United States.

This gives perspective to the challenges facing U.S. laboratories in respect to numerous competing priorities. For example, laboratories may be working to initially implement or upgrade probabilistic genotyping software in addition to considering other technological advancements such as next generation sequencing (NGS) that can supplement autosomal STR testing results with ancestry and phenotype information [[149], [150], [151]], Rapid DNA workflows in partnership with law enforcement agencies [152], and forensic genetic genealogy (FGG) [153]. In this context, it can be difficult to argue for the implementation of a “reconstructive” evaluation, that may be perceived to help serve a small percentage of cases, as it is competing for the already limited resources that labs need for current casework. The investment at the front end for developing a program in evaluations and reporting of results given activities is immense and would need to, at minimum, replicate a quality assurance program that is already in place in laboratories regarding other source-focused DNA testing methods.

#### 5.1.2 Research capabilities

Another impediment to the majority of publicly-funded U.S. laboratories is the absence of resources dedicated to research and development. This primarily impacts casework laboratories when it relates to new technologies and platforms related to DNA profile development and interpretation. Validation and implementation are costly in terms of personnel and time particularly when competing with requests for analysis and backlogs.

Not only would a research capability help expand the knowledge of DNA-TPPR related factors, but could enable case-specific experimentation, and support the development, validation, and implementation of evaluations of results given activity level propositions. Once this groundwork is laid, there will be a need to develop policies surrounding business-practices to include case acceptance and requirements for the evaluations.

#### 5.1.3 Case-acceptance and pre-assessment

There will certainly be a need for careful selection of cases as the available case information or research data may be insufficient to attempt an evaluation [34,82]. These resource constraints highlight the need for formalized policies and procedures within the laboratory regarding case acceptance that help establish the issues and questions prior to beginning testing following a CAI model [62,63]. Taylor & Kokshoorn provide examples for case acceptance and initiation in their textbook [82] and the NFI has standard operating procedures that they use to collect task-relevant information for reporting given activity level propositions and to guide the end-user through the process [154].

Submitting agencies need to be educated about the type of task-relevant information the laboratories need to properly assess and prioritize testing. Following the receipt of this information, a formal case pre-assessment can help the requester understand the potential value of the findings, for different outcomes, in respect to activity level propositions *before* the testing [9]. Pre-assessment has the added advantage of helping to mitigate bias or post hoc rationalization. For example, the ENFSI BPM [42] provides a pre-assessment table (see Table 5.) where the circumstances involve a female's accusation of digital penetration by a male individual.

**Table 5:** Case example of pre-assessment testing involves the fingers of accused.

Outcomes (E)	Pr(E|H_p_,I) if digital Penetration	Pr(E|H_d_,I) if social activities	Likelihood ratio Pr(E|H_p_,I)/Pr(E|H_d_,I)
Large quantity & full female profile	0.82	0.16	∼5
Small quantity & partial female profile	0.09	0.16	∼1
No female profile	0.09	0.68	∼1/7
Total	1	1	

In this example case pre-assessment involving an accusation of digital penetration by a male with a female complainant, three potential DNA-results outcomes are considered. A probability of obtaining the DNA result is assigned given each proposition (labeled “Hp” for prosecution's view and “Hd” for defense's view) is true which allows the calculation of a likelihood ratio. The probabilities presented here are for demonstrative purposes only and specific assignments would be drawn from in-house studies or published data sets. A benefit of this assessment is that it can be carried out prior to DNA testing, helping investigators and DNA scientists determine the best testing strategy. Table adapted from [42] with permission.

Case pre-assessment is useful not only for identifying cases in which activities are at issue, but also for providing timely and transparent information about the potential value of DNA testing. By considering the case questions at the outset, laboratories can assess the extent to which DNA testing is likely to help address those questions and prioritize items whose results have the potential to discriminate between competing source or activity level propositions.

Increasing casework demands, reluctance to validate new technologies, and limited research capacity within forensic laboratories can make implementation of new methods challenging. Evaluating biological findings given activity level propositions may further increase workload and turnaround times because these assessments often require more time than routine DNA comparison cases. However, a proactive case pre-assessment approach may offset some of this burden. By determining at case acceptance whether the proposed testing is likely to discriminate between the relevant propositions, laboratories may decline testing that is unlikely to add value and redirect resources toward cases in which DNA findings can meaningfully address disputed activities.

#### 5.1.4 Practitioner perspectives

In a limited survey in the U.S., over 70% of U.S. respondents (38/53 respondents) believed that a formalized approach to evaluative reporting given activity level propositions would be moderately to very useful to the jury; however, almost half of the respondents (46.3%) reported to be moderately uncomfortable with evaluations given activities [19]. The most common responses to anticipated issues related to education and training for scientists and legal professionals as well as challenges associated with court admissibility [19].

Approximately 88% (131/149 respondents) of global participants, a majority from Europe, either somewhat agreed, agreed, or strongly agreed with the view that a more formalized approach to evaluations given activities would be useful [20]. Global concerns with reporting given activities related to a lack of knowledge and education and the most common responses related to difficulties explaining propositions and LRs and concerns about acceptance in their legal system [20].

Anecdotally, the perception around these evaluations, whether formulaic or BN-based, is that they are extremely challenging and time consuming, which can be intimidating to many DNA casework practitioners.

### 5.2 Knowledge base and tools

Given the current resources of most forensic laboratories and the general absence of research capabilities, case-specific experimentation designed to help address questions in a case is likely not a feasible option. This continues to shift the burden of implementing evaluations given activity-level propositions toward the development of standardized, accessible knowledge bases. The need for such databases, analogous to allele frequency databases used to assign value to DNA comparisons, is not new and has been repeatedly emphasized in the literature [6,25,29,87,89]. Creating and maintaining such a database would be a substantial undertaking. Beyond identifying and collating the relevant literature, the information needs to be stored in a way that associates related studies into topics to support the extraction of probabilities of recovery given specified activities. Artificial intelligence may offer a means of identifying, collating, and organizing relevant TPPR publications^29^; however, important challenges remain. These include differential access to paid journal articles, potential copyright restrictions depending on how the model is deployed, and its use of published materials, as well as limitations in older publications that may not report sufficient detail regarding sample collection, testing, and interpretation methods to support reliable extraction or application of the data.

In a substantial effort towards the goal of one laboratory using another lab's data, the Recovery-Activity (ReAct) project aimed to collect data from a large number of casework laboratories and prepare generalized Bayesian networks (BNs) [30,102]. The study revealed that there was significant variability in the median recoveries of DNA (200 pg – 5 ng) between laboratories attributable to essentially unique methods per lab, to include sampling, quantitation, and PCR methods, which can impact the LR value assigned [30]. Taylor et al. developed a method to account for this variability in DNA quantities between laboratories when evaluating results given activity level propositions [92]. The ReAct project's work is ongoing to help reconcile the variability in quantity by applying an informative prior distribution as well as to continue to calibrate the models to ensure that the results are not misleading (i.e., a ground truth H_d_ scenario giving LR values greater than 1) [30]. Broderius and colleagues have been working on a multi-laboratory initiative primarily in Germany called “TransAct” aimed housing data collection from DNA-TPPR studies and found similar to ReAct that TPPR data are heavily impacted by laboratory-specific methods, particularly quantitative PCR methods [155].^30^

Understanding that an attempt to standardize DNA-TPPR data collection and reporting is paramount, FSI: Genetics released minimum publishing requirements to ensure that associated sample collection and methods are accessible to the wider community for use in experimentation and casework [103]. The requirements aim to facilitate the combination of data sets across multiple laboratories and improve reproducibility such that one laboratory can use the other lab's data for experimental or casework purposes and include requirements for probabilistic genotyping software, suitability for interpretation criteria, calculation of mixture ratios, and data that must be collected for each sample [103]. In a letter to the editor response to the minimum publishing requirements [156], the authors proposed templates for lab procedures and activity scenarios and suggested a mandatory checklist for both authors and reviewers to help ensure compliance with submission requirements.

Most methods for assigning value to findings given activity level propositions involve the use of software (e.g., Hugin, GeNIe) to construct BNs based on the case circumstances and data. As artificial intelligence (AI) and machine learning (ML) continue to develop, there may be areas that are ripe for consideration with this technology from reviewing and collating published literature and databases or building graphical interfaces to demonstrate how variables are connected. There are publications that provide templates for the scientists to examine [30,69,70]; however, it does not seem possible to stand-up a one size fits all approach as, for example, currently exists for DNA mixtures with probabilistic genotyping software which requires minimal user inputs (e.g., value(s) for number of contributors).

Continued progress towards the standardization and accessibility of data, multi-laboratory studies, and readily adaptable tools such as BN repositories should encourage the DNA community to move towards implementation by filling gaps in guidance for training, interpretation, reporting, and testimony. There is currently no consensus on the extent of specialized education or training needed and it is true that there are limited formal opportunities for training for forensic DNA professionals.^31^ Taylor & Kokshoorn's textbook discussed the education and training in section 11.1.4 [82] as well as the Netherlands Register for Court Experts (NRGD) where ‘DNA analysis and interpretation – activity level’ is recognized as a distinct competency.

### 5.3 Legal-related challenges

#### 5.3.1 Scientific literacy of the legal community

There is general agreement regarding the need for specialized education and training of the scientists who will engage in evaluations of results given activities [6,25,34,51,97,157] but it is important to emphasize that the investigators, attorneys, and judges will also need to engage in training. Work by de Roo and colleagues [158] found that criminal justice professionals (e.g., prosecutors, defense attorneys, judges) struggle to recognize flawed forensic reports and this is influenced by reports that originate from well-known and reputable institutions which are given less scrutiny.

A scientific push towards formalized, reported interpretations of results given activity level propositions will require the courts to engage with the methods in which DNA scientists give opinion in a more rigorous way just as Koehler and colleagues discuss in their paper about a scientific reinvention of the forensic sciences [159]. DNA testing technologies are perceived as generally reliable [160,161]; however, the limitations of current testing methods that help address source and their (in)ability to help address questions about DNA transfer do not appear well-understood in the courts outside of a few notable exceptions [162,163].

A national survey of judges by Garrett and colleagues regarding forensic science education indicates that there needs to be new efforts to expand scientific evidence education in the judiciary so that they can better comprehend the technical aspects as well as make better decisions about admissibility—judges also desire additional training in both law school and through continuing education opportunities [164]. In this study [165] Nir and Liu find that judges may over-rely on the prosecution's expert due to a combination of a lack of judiciary knowledge plus the inability of the defense attorney to effectively challenge admissibility or challenge expert witnesses. Albright [141] discusses that opinions should not exceed what is possible to conclude given the knowledge base and methods employed because a lay jury may not be able to identify false or exaggerated conclusions from complex evidence. Albright ultimately recommends stronger collaboration and understanding between the scientific and legal communities to help ensure that legal decisions are informed by trustworthy scientific knowledge [141].

#### 5.3.2 Can we reasonably assign the defense proposition?

Whether to assign a proxy defense proposition in the absence of a specific defense propositions has been discussed in the ENFSI guidelines [9], ISFG Part II [27], and Taylor & Kokshoorn's textbook [82] and remains a point of contention that bleeds into the legal arena where it can potentially impact a case in court if the assumptions regarding the defense proposition are not supported by case facts [39]. The ReAct paper considers this topic and warns that the scientist should be wary of assigning an alternative proposition on behalf of the defendant when there is simply a negation or no clear information about a specific transfer mechanism – such that a generic indirect transfer mechanism is considered through ‘social contact’ [30].

SWGDAM states that a laboratory implementing a formal approach to reporting would face practical issues with the adversarial U.S. criminal justice system since the defense has no legal obligation to provide their point of view, requiring the expert to speculate, and subject to evolving notice and disclosure this proposition could change [12]. The position statement also refers to significant legal issues with the admissibility of the findings, again, related to the formulation of the defense proposition and defense objections as discussed by Hunt [39].

Worth reiterating is that it is *never* the role of the scientist, under the principles of interpretation [[31], [32], [33]], to assert that a proposition (i.e., disputed case fact) is true, but to merely use it as a construct to adequately assess the DNA results just as has been done with questions of DNA sources since the inception of DNA analyses. Furthermore, the propositions that the scientist formulates are “sub-propositions” or those that may assist in answering one of many reasonable questions needing to be addressed in order for the factfinder to render the ultimate decision. Since activity level propositions are closer to the ultimate issue, it is no surprise that this can be an intimidating venture for scientists and legal professionals alike.

Hunt [39] discusses four circumstances where he believes that, legally in the U.S. system, an evaluation given activity level propositions could be performed due to the ability to assign a pair of competing propositions:

1. The alternative proposition is simply a negation of the prosecution's proposition.
2. The suspect provides a statement that includes specific information that allows the expert to formulate an alternative proposition.
3. The crime victim's statement or testimony provides specific information that allows the expert to formulate an alternative proposition; such as with circumstances where they may have interacted before the incident.
4. The defendant testifies at trial consistent with the prosecution expert's proxy alternative proposition.

Under the circumstances of a potentially vague alternative proposition such as some type of indirect transfer, the ReAct paper proposed the design of a proxy experiments that would aim to maximize the detection of a profile through the design of a reasonable experiment, such as the handshaking scenario examined in experiment 3.0 and argues that there would be no need for the defense to engage in specific methods of DNA transfer [30].

Finally, the U.S. scientific community may need to approach the formulation of propositions in a more user-friendly manner that is less focused on formality or accuracy while still adhering to transparency. The authors consider these issues to be best suited for pre-charging discussions or pre-trial litigation further reinforcing the need to author and disclose a report thereby helping to ensure that there is not a burden-shift towards the defense or the provision of information to the jury that may not align with the evidence to be presented during trial. In an assessment of his previous casework, Dr. Duncan Taylor relayed that the alternative propositions he considered often fell into the “negation” category which kept the propositions short and simple—additional background information and assumptions were stated separately in the report [166].

## 6 Concluding remarks

There is no shortage of barriers to overcome when assessing DNA results considering alleged activities whether the choice is to implement a formal reporting program or to train scientists to respond in accordance with the principles of interpretation and other professional codes of practice during testimony.

Assuming a consensus view within the U.S. legal community that aligns with Hunt [39], there remains ample opportunity for formal evaluations given activities described in Section 5.3.2. Such evaluations may be particularly important in circumstances where DNA results are a greater risk for being overvalued; for example, when relatively small quantities of DNA are detected, the cellular or body fluid origin of the DNA is unknown, or task-relevant information indicates a plausible transfer mechanism through recent interactions or cohabitation. For this reason, obtaining task-relevant information and performing case pre-assessment is crucial as it may significantly impact testing strategy and the value assigned to the biological findings. The Netherlands (NFI) provides one potential model, having focused on post-conviction cases where the DNA results may have been misunderstood or overvalued [122]. This emphasis on overvaluation does not disregard the converse risk of undervaluing DNA results in relation to activities, particularly when DNA is not detected despite a reasonable expectation that it would be present if the alleged activity occurred. Nevertheless, the authors consider overvaluation to be the more pressing concern. The high degree of trust placed in DNA evidence, reinforced by its prominent role in exonerations,^32^ may increase the risk for unwarranted conclusions about activities from results that, on their own, primarily address questions of source.

Widespread adoption of formal evaluations of DNA results considering alleged activities depends on the support of laboratory management that must facilitate progress towards closing gaps that have been consistently acknowledged, to include:

1) scientist training and competency;
2) investigator and attorney education and collaboration;
3) accessible and structured knowledge bases with DNA-TPPR data;
4) standards development in training, interpretation, reporting, and testimony;
5) availability of interpretation tools such as a collection of Bayesian network templates; and
6) practitioner apprehension due to the learning curve and legal acceptance; and
7) operational challenges within U.S. laboratories like stretched resources, volume of backlogged casework, case acceptance and assessment approaches, and formulating defense propositions.

There are still common misconceptions about what the DNA comparison means in a case—equally important is the consideration of what the absence of DNA results means [167] as well as the knowledge that the mere presence of a DNA profile (aligning with a person of interest) does *not* automatically imply an associated activity [10,27,35]. A warning to prevent the translation of a DNA comparison LR value to issues about the activities, however, may be inadequate in certain cases—and it is in these circumstances that the scientist should endeavor to provide guidance. Biedermann and colleagues called for scientists to help with a readjustment of perspective—to ensure that forensic DNA results are meaningfully used in the legal process, scientists must first work on improving their knowledge and understanding of additional DNA-TPPR factors that impact interpretation [35]. It appears that scientists understand that considering transfer is an important aspect of interpretation of DNA traces [19,20,168]; however, there is apprehension to implement interpretation and reporting methods whether due to (a lack of) education, anticipated legal challenges, or the complexity of the evaluations.

Where do we go from here? The legal history of DNA analysis [169] has shown us that with anything “new” in forensic science, there will be challenges to admissibility in addition to addressing the scientific methodology gaps. But the community must start somewhere and the first step should be the education of the scientists—once that has been more widely achieved, they can collaborate with other parties in the criminal justice system to spread that knowledge. There has been a consistent offering of training workshops designed to be 4 to 5 days in length aimed at providing the participants with extensive user training for STRmix™^33^ and there is no reason why something similar could not be accomplished in relation to evaluations of biological results given activity level propositions. The course could take a blended approach of 40 hours of in-person time with additional hours of self-directed practice and mentorship. Once this training has been taken by the scientists, they will have the necessary basis to 1) decide if moving in this direction is justified and 2) create the quality assurance framework necessary for any new forensic discipline.

In the meantime, ongoing projects like the ENFSI/European Union sponsored Recovery Activity (ReAct) interlaboratory studies [30] will continue to assess the reproducibility of results, how the inter-examiner variability can be accounted for, and how this impacts the resulting LR—to include rates of misleading evidence—which will help to solidify the legitimacy and credibility of the methods [170,171] thereby inspiring end-user and public trust. Once these areas are well characterized, the community will inevitably put in the effort to establish and adopt standards specific to reporting of DNA results given activities.

Finally, the scientific and legal communities must determine the appropriate scope—whether general or case-specific—of expert opinion testimony concerning DNA transfer. Developing a responsible and scientific approach will require collaboration and careful consideration of the scientific validity of the methods used, transparency regarding the assumptions and bases underlying any opinion, effective communication of limitations, and, ultimately, whether the evaluation will assist the decision-maker. At present, DNA experts may be required, often with limited preparation and under the pressures of trial, to choose between offering little guidance of what the DNA findings mean in the context of the case and providing an opinion that has not been fully documented, supported by a case-specific evaluation, or reviewed, assuming the expert is trained to offer it. In some circumstances, the DNA expert's knowledge may be crucial to prevent the significance of a DNA comparison from being misunderstood, particularly when individuals cohabitate or routinely share tested items, as illustrated by the Meredith Kercher case [44]. In other circumstances, an opinion offered without sufficient time, ambiguous or missing case information, or appropriate scientific support may mischaracterize the value of the DNA findings. This places the DNA expert in a difficult position and highlights the need for more robust policies and procedures governing the qualifications, preparation, review, and presentation of such testimony.

## CRediT authorship contribution statement

**Jarrah R. Kennedy:** Writing – review & editing, Writing – original draft, Conceptualization. **Christophe Champod:** Writing – review & editing, Writing – original draft, Supervision.

## Declaration of competing interest

The authors declare the following financial interests/personal relationships which may be considered as potential competing interests: Jarrah Kennedy reports administrative support was provided by Texas Forensic Science Commission. Jarrah Kennedy reports a relationship with Texas Forensic Science Commission that includes: employment. The corresponding author is the current Scientific Program Coordinator for the Texas Forensic Science Commission (TFSC); however, the statements and opinions contained herein do not necessarily reflect those of the TFSC staff or of the Commissioners and should not be understood as a TFSC position statement. There are no other competing or financial interests to declare. If there are other authors, they declare that they have no known competing financial interests or personal relationships that could have appeared to influence the work reported in this paper.

## Footnotes

1 The ability to interpret complex mixtures, and give value to DNA comparisons, continues to expand with the implementation of probabilistic genotyping software.

2 Formal evaluations are defined here just as SWGDAM defines formal activity level reporting which refers to an evaluation of the biological findings given activity level propositions performed in advance of trial, using a Bayesian logical framework, technically reviewed, and provided to relevant parties in a laboratory report.

3 [2009] EWCA Crim 2698, [2010] 1 Cr App R 23.

4 [2010] EWCA Crim 1085.

5 Decision-maker here is used to encompass the following parties: investigator, prosecuting attorney, defense attorney, judge, or jury.

6 Competing activity level propositions, by definition, contain language that is closer to the offense such as “stab,” “digital penetration” or “vaginal-penile intercourse” which may also be a trigger for legal professionals.

7 POI stands for “person of interest” and relates to any individual, not just a potential suspect.

8 Terrell Gills was associated to the case following a partial DNA ‘match’ via the CODIS database. He plead not guilty and was acquitted at trial due in large part to the introduction of video evidence and information about the conviction of another individual for two other related crimes at Dunkin Donuts, see https://www.nytimes.com/2017/05/16/nyregion/can-dna-evidence-be-too-convincing-an-acquitted-man-thinks-so.html [accessed June 26, 2025].

9 *See* 37 Tex. Admin. Code § 651.219.

10 *See* TEX. CODE OF CRIM. PROC. art. 38.01 § 4-a(a)(2): Texas law defines the term “forensic analyst” as “a person who on behalf of a crime laboratory [accredited by the Commission] technically reviews or performs a forensic analysis or draws conclusions from or interprets a forensic analysis for a court or crime laboratory.

11 *See* TEX. CODE OF CRIM. PROC. art. 38.35 § (a)(1): Texas law defines the term “forensic analysis” as a medical, chemical, toxicological, ballistic, or other examination or test performed on physical evidence, including DNA evidence, for the purpose of determining its connection to a criminal action.

12 *See* TEX. CODE OF CRIM. PROC art. 38.01(4)(b) holds that a person must be licensed to act as a forensic analyst. *See* 37 Tex. Admin. Code § 651.216(a)(1)-(4) for potential actions to be taken against a license holder.

13 Per Dr. Kalafut's response to complaint 23.67 “I have given accepted testimony on activity level propositions since at least 2003, and that specific testimony was upheld by an appellate court in *United States vs. Albert Hill*.*” See Final Report on Complaint No. 23.*67 by *the Texas Forensic Science Commission, July 26, 2024*.

14 **Consensus recommendation 15:** A report shall be issued wherever practical. The report shall include, at a minimum, the case information relied upon, the propositions used, an assessment of the LR in a numerical form. A verbal qualifier is optional. The report shall include all relevant traceability information. The report should invite the defense to have input.

15 There are many other verbal qualifiers or so-called “scales” that can be chosen including examples provided by ENFSI Guideline 2015 and Marquis et al. 2016 Discussion on how to implement a verbal scale in a forensic laboratory: Benefits, pitfalls and suggestions to avoid misunderstandings.

16 Not discussed here is the “weak evidence effect” when presenting “smaller” likelihood ratios that may fall into limited or weak support verbal equivalent categories such as would be commonly observed with evaluations of results given activity level propositions where when the end-user misinterprets a verbal qualifier for “weak” support for one proposition versus the other as support for the “other” proposition. This effect has not been examined or replicated since first reported by Martire in 2013-14. However, such tendency lends even more support for reporting and communicating numerical LRs.

17 Consistency: to give equal weight to evidence of equal strength; Ability: to be able to infer new information from the evidence; Sensitivity: to give more/less weight to evidence of greater/lesser strength; Orthodoxy: to update beliefs in line with Bayesian normative expectations; Coherence: to treat evidence in a logical and rational manner.

18 Section 5.2. “Sensitivity is assigning greater weight to evidence of greater value, and lesser weight to evidence of lesser value.”

19 Section 5.3. “Orthodoxy is used in the sense of compliance with or adherence to normative expectations, i.e., orthodoxy is updating beliefs in a manner that is consistent with the normative expectations derived using Bayes' theorem.”

20 Section 5.4. “Coherence is responding to evidence in a logical manner.” “This definition excludes a range of potentially ‘incoherent’ lay responses to statistical statements that are incompatible with genuine comprehension such as the prosecutor's and Defense Attorney's Fallacies (citations removed).”

21 Morrison and colleagues write that random-match probabilities are in conflict with data that have within-source variability (the numerator is not always one) and that verbal likelihood ratios and verbal strength-of-support statements have no intrinsic meaning and that they rely on a range of numerical LRs to which they are associated.

22 *R* v *T* [2010] EWCA Crim 2439, [2011] 1 Cr App R 9.

23 The Parts are available from the ISO website (https://www.iso.org/standards.html).

24 *Brown v. Farwell, 525 F.3d 787 (9*th *Cir. 200), cert. granted sub nom. McDaniel v. Brown, 129 S.Ct. 1038 (2009), 173-176, 286n16:* DNA expert Romero committed the transposition fallacy by conflating the RMP (1 in 3 million) with the source probability by stating that there was a 99.99967% chance that the DNA in the victim's underwear was from the defendant. The U.S. court of appeals for the Ninth Circuit granted relief because absent the faulty DNA testimony, there was not sufficient evidence to sustain his conviction. The court pointed to particularly weak non-DNA evidence and the “jury's consideration of false, but highly persuasive, evidence.”; *McDaniel vs. Brown 558 U. S. ____ (2010)* would weigh on this case and reverse the Ninth Circuit's decision—the court would need to consider of ineffective assistance of counsel claims. Reiterated fair and reliable presentation of evidence due to its persuasive power; however, the court felt that the non-DNA evidence supported the jury's guilty verdict.

25 There are several footnotes from this section of Hunt's paper are not duplicated here that discuss the role of expert witnesses, FED R. EVID 702 with respect to explanations, and a scientific reference about the distinction between explanations and propositions.

26 https://bja.ojp.gov/doc/fact-sheet-dna-cebr.pdf [accessed November 25, 2025].

27 Texas State Government Code Sub-Chapter B-1. Analysis of Sexual Assault of other sex offense: § 420.042(c).

28 Texas Code of Criminal Procedure Article 38.43(i)(j)(k).

29 Janet Stacey (The University of Auckland/PHF Science, New Zealand) presented “Assessing the Data Available for Activity-Level Evaluations” at the 31st Congress of the International Society for Forensic Genetics (ISFG) in Montreal: https://isfg2026.ca/program/congress-program/conference?tx_congres_pi_principal%5Baction%5D=show&tx_congres_pi_principal%5Bcontroller%5D=Items&tx_congres_pi_principal%5Bitem%5D=52&cHash=095875901dfd15967806ea4bd749eb41 [accessed August 27, 2026].

30 Kathrin Broderius (Institute of Legal Medicine, University Hospital of Cologne, Germany) presented “Methods Matter: On the Impact of Non-transferential Variables on Reported Data of Studies on Nucleic Acid Transfer, Prevalence, Persistence and Recovery (TPPR)”” at the 31st Congress of the International Society for Forensic Genetics (ISFG) in Montreal: https://isfg2026.ca/program/congress-program/conference?tx_congres_pi_principal%5Baction%5D=show&tx_congres_pi_principal%5Bcontroller%5D=Items&tx_congres_pi_principal%5Bitem%5D=51&cHash=33b43c3fc430f5ac2bc674f594fb3f63.

31 The authors know of only one specialized and intensive education course through the University of Lausanne's Faculty of Law, Criminal Justice and Public Administration (FDCA) which is on-line, covering 8 modules over 13 months with approximately 4 h of work per week. See https://www.formation-continue-unil-epfl.ch/formation/advanced-dna-interpretation/[accessed November 25, 2025].

32 See DNA Exonerations in the United States (1989 – 2020): https://innocenceproject.org/dna-exonerations-in-the-united-states/[accessed August 27, 2026].

33 STRmix™ Training and Events: https://www.strmix.com/training-and-events [accessed February 7, 2026].
