# Supplementary material for: Current state: Assessing DNA results considering transfer
Source: Forensic Sci Int Synerg. 2026 Sep 16;13:100733. doi: 10.1016/j.fsisyn.2026.100733 (PMC13598168; doi:10.1016/j.fsisyn.2026.100733)
Supplement: Supplementary file 1 [file mmc1.docx]

# Supplemental materials

The following tables are not meant to be exhaustive but rather a starting point when looking for information about evaluations of findings given activity level propositions. Supplementary Table A. is a compilation of DNA-TPPR factors. Tables B, C, and D categorize specific articles related to the foundations of reasoning in forensic DNA interpretation, available standards and guidance documents, and resources related to DNA-TPPR factors and evaluations given activity level propositions, respectively.

## Supplemental Table A. Summary of DNA Transfer, Persistence, Prevalence, Recovery (TPPR) and Background

| Transfer | Persistence | Prevalence | Recovery | Background |
| --- | --- | --- | --- | --- |
| **Methods of how (DNA) can be left behind:**   - **Direct (primary)** - **Indirect (secondary, tertiary, quaternary)** - **Bi-directional (two-way)** | **Retention of (biological) material after deposition till recovery** | **DNA that is unrelated to incident and expected to be present** | **Collection of material and all laboratory methods to detect and interpret DNA profiles** | **Unknown DNA present for unknown reasons** |
| *Who* transferred?   - Shedder status - Age - Sex - Skin conditions - Previous actions | Environmental exposure:   - UV - Heat - Moisture - Chemicals | Owner DNA (e.g., steering wheel, items in home).  Individual: nature of material, body location.  Often basis for conditioning of profiles. | Sampling methods: swab(s), tape lift, cutting, and scraping.  DNA extraction methods, efficiencies, volumes.  DNA quantitation methods. | Material that cannot be traced backed to any reference material or specific mechanisms for deposition. |
| *What* was transferred?   - Nature of material (e.g., blood, semen) - Wet or dry - Nature of touch deposits | What happened after the activity of interest?   - Time between incident and collection - Hand washing, shower | NOT background DNA  Importance of collecting references from individuals with access to scenes | Amplification parameters and sensitivity:   - Kit, DNA input, cycles   Detection platform:   - Injection time duration - Voltage | Non-self-DNA on the individual body parts (e.g., fingernails, skin surfaces) or objects (e.g., clothing, tools, weapons). |
| *How* transferred? (activity)   - Manner of contact - Intensity of contact - Duration of contact - Area of contact | How was the item handled during the forensic process?   - Collection - Handling - Storage | May need to sample from other areas of same item; other items in contact; similar items; and the broader scene. | Analysis parameters:   - Analytical threshold - Stutter thresholds - Peak editing   Interpretation methods:   - Binary or PGS - Compatibility: presence/absence, allele counting, LR values | Unexplained contributors to items or mixtures could represent alternative contributors under a defense proposition and should not be ignored. |
| *From/to* what (object)?   - Surface texture/material - Nature of intermediate surface (indirect) |  |  | See ReAct and TransAct projects – variability between labs in the amount of DNA recovered. | May be able to use pre-experimental data or control group data from studies. |

Table inspired by Gosch and Courts [1], Gill and colleagues [2], Taylor & Kokshoorn [3], and Broderius presented “Methods Matter: On the Impact of Non‑transferential Variables on Reported Data of Studies on Nucleic Acid Transfer, Prevalence, Persistence and Recovery (TPPR)” at the 31^st^ Congress of the International Society for Forensic Genetics (ISFG) in Montreal <https://isfg2026.ca/program/congress-program/conference?tx_congres_pi_principal%5Baction%5D=show&tx_congres_pi_principal%5Bcontroller%5D=Items&tx_congres_pi_principal%5Bitem%5D=51&cHash=33b43c3fc430f5ac2bc674f594fb3f63>

## Supplemental Table B. Summary of Key Citations—DNA Interpretation Framework

| Title and Citation |  | Topic |
| --- | --- | --- |
| A Logical Framework for Forensic DNA Interpretation [4] |  | Overarching review article |
| A Model for Case Assessment and Interpretation [5];  Case Assessment and Interpretation [6];  The Development of Case Assessment and Interpretation (CAI) in Forensic Science [7];  Case Assessment and Interpretation of Expert Evidence: Guidance for Judges, Lawyers, Forensic Scientists and Expert Witnesses [8] |  | Case Assessment and Interpretation (CAI) |
| Avoiding the Transposed Conditional [9];  A Model for Case Assessment and Interpretation [10];  The Scientist and the Scale of Justice [11];  The Impact of the Principles of Evidence Interpretation on the Structure and Content of Statements [12] |  | Principles of Interpretation and desiderata |
| A Hierarchy of Propositions: Deciding Which Level to Address in Casework [13];  More in the Hierarchy of Propositions: Exploring the Distinction Between Explanations and Propositions [14];  Whose DNA is this? How relevant a question? (a note for forensic scientists) [15];  The importance of distinguishing information from evidence/observations when formulating propositions [16];  A Practical Guide for the Formulation of Propositions in the Bayesian Approach to DNA Evidence Interpretation in an Adversarial Environment [17] |  | Hierarchy of Propositions and formulating propositions |
| The Nature of Forensic Science Opinion – a Possible Framework to Guide Thinking and Practice in Investigations and in Court Proceedings [18] |  | Investigative and Evaluative roles |
| DNA commission of the International society for forensic genetics: Assessing the value of forensic biological evidence - Guidelines highlighting the importance of propositions: Part I: evaluation of DNA profiling comparisons given (sub-) source propositions [19] |  | Guideline specific to (sub)-source level propositions |

## Supplemental Table C. Index of standards, guidelines, and other documents: evaluative reporting given activities

| Document Title | Year |
| --- | --- |
| Standards for the formulation of evaluative forensic science expert opinion [20] | 2009 |
| European Network of Forensic Science Institutes (ENFSI) Guideline for Evaluative Reporting in Forensic Science [21] | 2015 |
| An introductory guide to evaluative reporting [22] | 2017 |
| DNA Commission of the ISFG – Assessing the value of forensic biological evidence - Guidelines highlighting the importance of propositions. Part II: Evaluation of biological traces considering activity level propositions [2] | 2020 |
| Development of evaluative opinions: codes of practice and conduct for forensic service providers FSR-118-C Issue 1 [23] | 2021 |
| European Network of Forensic Science Institutes (ENFSI) Best Practice Manual for Human Forensic Biology and DNA Profiling [24] | 2022 |
| Forensic DNA Interpretation and Human Factors: Improving Practice Through a Systems Approach [25] | 2024 |
| Final Report on Complaint No. 23.67; Tiffany Roy; (Timothy Kalafut PhD; Evaluation of Biological/DNA Results Given Activity Level Propositions) [26] | 2024 |
| DNA Mixture Interpretation: A NIST Scientific Foundation Review [27] | 2024 |
| Reporting the value of biological findings given activity level propositions: A discussion of published recommendations [28] | 2025 |
| ISO 21043-3:2025 Forensic Sciences – Part 3: Analysis^[1]^  ISO 21043-4:2025 Forensic Sciences – Part 4: Interpretation^[2]^  ISO 21043-5:2025 Forensic Sciences – Part 5: Reporting^[3]^ | 2025 |
| Guidance: Forensic Science Activities Interpretation and communication FSR-GUI-0004 | 2026 |

## Supplemental Table D. Categorization of key publications for evaluations given activity level propositions

| Title and Citation | Topic |
| --- | --- |
| A Quantitative Theory for Interpreting Transfer Evidence in Criminal Cases [29];  Interpreting Small Quantities of DNA: The Hierarchy of Propositions and the Use of Bayesian Networks [30];  Bayesian networks and probabilistic reasoning about scientific evidence when there is a lack of data [31]  Bayesian networks for evaluating forensic DNA profiling evidence: A review and guide to literature [32];  Bayesian Networks for Probabilistic Inference and Decision Analysis in Forensic Science [33];  Evaluation of forensic genetics findings given activity level propositions: A review [34];  More on the question 'When does absence of evidence constitute evidence of absence?' How Bayesian confirmation theory can logically support the answer [35];  Forensic DNA Trace Evidence Interpretation Activity Level Propositions and Likelihood Ratios [3] | Approaches to evaluation (Bayesian inference, Bayesian Networks) |
| Helping to distinguish primary from secondary transfer events for trace DNA [36];  Transfer and persistence of non-self DNA on hands over time: Using empirical data to evaluate DNA evidence given activity level propositions [37];  A template for constructing Bayesian networks in forensic biology cases when considering activity level propositions [38];  Using Bayesian networks to track DNA movement through complex transfer scenarios [39];  The use of Bayesian Networks and simulation methods to identify the variables impacting the value of evidence assessed under activity level propositions in stabbing cases [40];  A collection of idioms for modeling activity level evaluations in forensic science [41];  Driver or passenger? Use of a Bayesian network for the evaluation of DNA results in a fatal car accident [42] | Templates and publications where BNs used to assess the findings |
| Activity level DNA evidence evaluation: On propositions addressing the actor or the activity [43];  Structuring cases into propositions, assumptions, and undisputed case information [44]; | Structuring case information and assigning propositions |
| Using sensitivity analyses in Bayesian Networks to highlight the impact of data paucity and direct future analyses: a contribution to the debate on measuring and reporting the precision of likelihood ratios [45];  An LR framework incorporating sensitivity analysis to model multiple direct and secondary transfer events on skin surface [46];  A practical treatment of sensitivity analyses in activity level evaluations [47]; | Sensitivity Analyses |
| Sharing data on DNA transfer, persistence, prevalence and recovery: Arguments for harmonization and standardization [48];  Interpretation continues to be the main weakness in criminal justice systems: Developing roles of the expert witness and court [49]  On DNA transfer: The lack and difficulty of systematic research and how to do it better [1];  Evaluating forensic DNA evidence: Connecting the dots [50];  The occurrence and genesis of transfer traces in forensic science: a structured knowledge database [51];  DNA Transfer in Forensic Science: Recent Progress towards Meeting Challenges [52];  The ReAct project: Analysis of data from 23 different laboratories to characterise DNA recovery given two sets of activity level propositions [53];  Editorial: The ReAct project: Bayesian networks for assessing the value of the results given activity level propositions [54];  Current state and barriers to global adoption of forensic evaluative reporting of findings given activity-level propositions [55];  Minimum FSI: Genetics requirements for publishing data on DNA transfer and recovery, given activities [56];  Letter to the Editor Response to "Minimum FSI: Genetics requirements for publishing data on DNA transfer and recovery, given activities" [57];  Preprint not peer reviewed yet: Methods matter: On the impact of non-transferential variables on reported data of studies on nucleic acid transfer, prevalence, persistence and recovery (TPPR) [58] | Development of a knowledge-base: standardization of DNA-TPPR collection and storage |
| Accounting for inter-laboratory DNA recovery data variability when performing evaluations given activities [59] | Accounting for Variability in DNA recovery |
| Need for dedicated training, competency assessment, authorisations and ongoing proficiency testing for those addressing DNA transfer issues [60];  Problematic reporting in DNA cases: the need for accredited formats and certified reporting competence [61];  Evaluating forensic DNA evidence: Connecting the dots [50];  DNA Transfer in Forensic Science: Recent Progress towards Meeting Challenges [52];  Final Report on Complaint No. 23.67; Tiffany Roy; (Timothy Kalafut PhD; Evaluation of Biological/DNA Results Given Activity Level Propositions) [26];  Current state and barriers to global adoption of forensic evaluative reporting of findings given activity-level propositions [55] | The need for adequate training, education in interpretation and reporting |
| Birgitte Tengs case: analysis and the wider implications for evaluation of DNA evidence given activities [62];  A comparison of graphical methods using the case of the murder of Meredith Kercher as an example [63];  Bayesian networks and dissonant items of evidence: A case study [64];  The importance of evaluating findings given activity level propositions in order to avoid misleading evidence [65] | Importance of evaluations given activity level propositions using specific cases |
| Forensic trace DNA: A review [66];  DNA transfer: Review and implications for casework [67];  DNA transfer in forensic science: A review [68];  A review of trace "Touch DNA" deposits: Variability factors and an exploration of cellular composition [69]  DNA Transfer in Forensic Science: Recent Progress towards Meeting Challenges [52];  Indirect DNA Transfer and Forensic Implications: A Literature Review [70];  DNA Mixture Interpretation: A NIST Scientific Foundation Review [27];  The Transfer, Prevalence, Persistence, and Recovery of DNA from Body Areas in Forensic Science: A Review [71] | Review articles about DNA-TPPR factors |

# References

[1]	Gosch A, Courts C. On DNA transfer: The lack and difficulty of systematic research and how to do it better. Forensic Science International: Genetics. 2019;40:24-36.

[2]	Gill P, Hicks T, Butler JM, Connolly E, Gusmão L, Kokshoorn B, et al. DNA commission of the International society for forensic genetics: Assessing the value of forensic biological evidence - Guidelines highlighting the importance of propositions. Part II: Evaluation of biological traces considering activity level propositions. Forensic Science International: Genetics. 2020;44:102186.

[3]	Taylor D, Kokshoorn B. Forensic DNA Trace Evidence Interpretation Activity Level Propositions and Likelihood Ratios. 1st Edition ed. Boca Raton, FL: CRC Press; 2023.

[4]	Hicks T, Buckleton J, Castella V, Evett I, Jackson G. A Logical Framework for Forensic DNA Interpretation. Genes. 2022;13(6):957.

[5]	Cook R, Evett IW, Jackson G, Jones PJ, Lambert JA. A model for case assessment and interpretation. Sci Justice. 1998;38(3):151-6.

[6]	Jackson G, Jones PJ. Case Assessment and Interpretation. In: Moenssens A, Jamieson A, editors. Wiley Encyclopedia of Forensic Science. 2. Chichester, UK: John Wiley & Sons; 2009. p. 483-96.

[7]	Jackson G. The Development of Case Assessment and Interpretation (CAI) in Forensic Science: D. Phil University of Abertay Dundee; 2011.

[8]	Jackson G, Aitken C, Roberts P. Case Assessment and Interpretation of Expert Evidence: Guidance for Judges, Lawyers, Forensic Scientists and Expert Witnesses. London: The Royal Statistical Society; 2014.

[9]	Evett IW. Avoiding the Transposed Conditional. Science & Justice. 1995;35(2):127-31.

[10]	Cook R, Evett IW, Jackson G, Jones PJ, Lambert JA. A Model for Case Assessment and Interpretation. Science & Justice. 1998;38(3):151-6.

[11]	Jackson G. The Scientist and the Scale of Justice. Science & Justice. 2000;40:81-5.

[12]	Evett IW, Jackson G, Lambert JA, McCrossan S. The Impact of the Principles of Evidence Interpretation on the Structure and Content of Statements. Science & Justice. 2000;40(4):233-9.

[13]	Cook R, Evett IW, Jackson G, Jones PJ, Lambert JA. A Hierarchy of Propositions: Deciding Which Level to Address in Casework. Science & Justice. 1998;38(4):231-40.

[14]	Evett IW, Jackson G, Lambert JA. More in the Hierarchy of Propositions: Exploring the Distinction Between Explanations and Propositions. Science & Justice. 2000;40(1):3-10.

[15]	Taroni F, Biedermann A, Vuille J, Morling N. Whose DNA is this? How relevant a question? (a note for forensic scientists). Forensic Science International: Genetics. 2013;7(4):467-70.

[16]	Hicks T, Biedermann A, de Koeijer JA, Taroni F, Champod C, Evett IW. The importance of distinguishing information from evidence/observations when formulating propositions. Science & Justice. 2015;55(6):520-5.

[17]	Gittelson S, Kalafut T, Myers S, Taylor D, Hicks T, Taroni F, et al. A Practical Guide for the Formulation of Propositions in the Bayesian Approach to DNA Evidence Interpretation in an Adversarial Environment. Journal of Forensic Sciences. 2015;61(1):186-95.

[18]	Jackson G, Jones S, Booth G, Champod C, Evett IW. The Nature of Forensic Science Opinion – a Possible Framework to Guide Thinking and Practice in Investigations and in Court Proceedings. Science & Justice. 2006;46(1):33-44.

[19]	Gill P, Hicks T, Butler JM, Connolly E, Gusmão L, Kokshoorn B, et al. DNA commission of the International society for forensic genetics: Assessing the value of forensic biological evidence - Guidelines highlighting the importance of propositions: Part I: evaluation of DNA profiling comparisons given (sub-) source propositions. Forensic Science International: Genetics. 2018;36:189-202.

[20]	Association of Forensic Science Providers. Standards for the formulation of evaluative forensic science expert opinion. Science & Justice. 2009;49(3):161-4.

[21]	Willis SMM, L.; McDermott, S.; O’Donnell, G.; Barrett, A.; Rasmusson, B.; Hoglund, T.; Nordgaard, A.; Berger, C.; Sjerps, M.; Molina, J.J.L.; Zadora, G.; Aitken, C.; Lovelock. T.; Lunt, L.; Champod, C.; Biedermann, A.; Hicks, T.; Taroni, F. ENFSI Guideline for Evaluative Reporting in Forensic Science. Dublin: European Network of Forensic Science Institutes; 2015.

[22]	National Institute of Forensic Science Australia and New Zealand. An introductory guide to evaluative reporting. National Institute of Forensic Science Australia and New Zealand; 2017.

[23]	The Forensic Science Regulator in partnership with the Chartered Society of Forensic Sciences and the Royal Statistical Society. Development of evaluative opinions: codes of practice and conduct for forensic service providers. Brimingham: Forensic Science Regulator; 2021. Contract No.: FSR-C-118.

[24]	European Network of Forensic Science Institutes. Best practice manual for human forensic biology and DNA profiling. Version 01. ENFSI-DNA-BPM-03. Wiesbaden: European Network of Forensic Science Institutes; 2022.

[25]	Expert Working Group on Human Factors in Forensic DNA Interpretation. Forensic DNA Interpretation and Human Factors: Improving Practice Through a Systems Approach. National Institute of Standards and Technology; 2024.

[26]	Texas Forensic Science Commission. Final Report on Complaint No. 23.67; Tiffany Roy; (Timothy Kalafut PhD; Evaluation of Biological/DNA Results Given Activity Level Propositions). Austin, TX; 2024 July 26.

[27]	Butler JM, et al. DNA Mixture Interpretation: A NIST Scientific Foundation Review. Gaithersburg, MD: National Institute of Standards and Technology; 2024. Contract No.: NIST IR 8351. <https://doi.org/10.6028/NIST.IR.8351>.

[28]	Buckleton J, Taylor D, Allen PS, Curran J, Kalafut T. Reporting the value of biological findings given activity level propositions: A discussion of published recommendations. Journal of Forensic Sciences. 2025;70:1670-86.

[29]	Evett IW. A Quantitative Theory for Interpreting Transfer Evidence in Criminal Cases. Applied Statistics. 1984;33(1):25-32.

[30]	Evett IW, Gill PD, Jackson G, Whitaker J, Champod C. Interpreting Small Quantities of DNA: The Hierarchy of Propositions and the Use of Bayesian Networks. Journal of Forensic Sciences. 2002;47(3):520-30.

[31]	Biedermann A, Taroni F. Bayesian networks and probabilistic reasoning about scientific evidence when there is a lack of data. Forensic Science International. 2006;157(2-3):163-7.

[32]	Biedermann A, Taroni F. Bayesian networks for evaluating forensic DNA profiling evidence: A review and guide to literature. Forensic Science International: Genetics. 2012;6(2):147-57.

[33]	Taroni F, Biedermann A, Bozza S, Garbolino P, Aitken C. Bayesian Networks for Probabilistic Inference and Decision Analysis in Forensic Science. 2nd ed. Barnett V, editor. Chichester: John Wiley & Sons, Ltd; 2014.

[34]	Taylor D, Kokshoorn B, Biedermann A. Evaluation of forensic genetics findings given activity level propositions: A review. Forensic Science International: Genetics. 2018;36:34-49.

[35]	Taroni F, Bozza S, Hicks T, Garbolino P. More on the question 'When does absence of evidence constitute evidence of absence?' How Bayesian confirmation theory can logically support the answer. Forensic Science International. 2019;301:e59-e63.

[36]	Taylor D, Biedermann A, Samie L, Pun K-M, Hicks T, Champod C. Helping to distinguish primary from secondary transfer events for trace DNA. Forensic Science International: Genetics. 2017;28(May):155-77.

[37]	Szkuta B, Ballantyne KN, Kokshoorn B, van Oorschot RAH. Transfer and persistence of non-self DNA on hands over time: Using empirical data to evaluate DNA evidence given activity level propositions. Forensic Science International: Genetics. 2018;33:84-97.

[38]	Taylor D, Biedermann A, Hicks T, Champod C. A template for constructing Bayesian networks in forensic biology cases when considering activity level propositions. Forensic Science International: Genetics. 2018;33:136-46.

[39]	Taylor D, Samie L, Champod C. Using Bayesian networks to track DNA movement through complex transfer scenarios. Forensic Science International: Genetics. 2019;42:69-80.

[40]	Samie L, Champod C, Taylor D, Taroni F. The use of Bayesian Networks and simulation methods to identify the variables impacting the value of evidence assessed under activity level propositions in stabbing cases. Forensic Science International: Genetics. 2020;48:102334.

[41]	Vink M, Sjerps MJ. A collection of idioms for modeling activity level evaluations in forensic science. Forensic Science International: Synergy. 2023:100331.

[42]	Samie L, Champod C, Hicks T, Delemont S, Castella V. Driver or passenger? Use of a Bayesian network for the evaluation of DNA results in a fatal car accident. Forensic Science International: Genetics. 2025;74:103166.

[43]	Kokshoorn B, Blankers BJ, de Zoete J, Berger CEH. Activity level DNA evidence evaluation: On propositions addressing the actor or the activity. Forensic Science International. 2017;278:115-24.

[44]	Taylor D, Kokshoorn B, Hicks T. Structuring cases into propositions, assumptions, and undisputed case information. Forensic Sci Int Genet. 2020;44:102199.

[45]	Taylor D, Hicks T, Champod C. Using sensitivity analyses in Bayesian Networks to highlight the impact of data paucity and direct future analyses: a contribution to the debate on measuring and reporting the precision of likelihood ratios. Science & Justice. 2016;56(5):402–10.

[46]	Gill P, Bleka Ø, Roseth A, Fonneløp AE. An LR framework incorporating sensitivity analysis to model multiple direct and secondary transfer events on skin surface. Forensic Science International: Genetics. 2021;53:102509.

[47]	Taylor D, Kokshoorn B, Champod C. A practical treatment of sensitivity analyses in activity level evaluations. Forensic Science International. 2024;355:111944.

[48]	Kokshoorn B, Aarts LHJ, Ansell R, Connolly E, Drotz W, Kloosterman AD, et al. Sharing data on DNA transfer, persistence, prevalence and recovery: Arguments for harmonization and standardization. Forensic Science International: Genetics. 2018;37:260-9.

[49]	Gill P. Interpretation continues to be the main weakness in criminal justice systems: Developing roles of the expert witness and court. WIREs Forensic Science. 2019;1(2):e1321.

[50]	Meakin GE, Kokshoorn B, van Oorschot RAH, Szkuta B. Evaluating forensic DNA evidence: Connecting the dots. WIREs Forensic Science. 2020:e1404.

[51]	Cadola L, Charest M, Lavallée C, Crispino F. The occurrence and genesis of transfer traces in forensic science: a structured knowledge database. Canadian Society of Forensic Science Journal. 2021;54(2):86-100.

[52]	van Oorschot RAH, Meakin GE, Kokshoorn B, Goray M, Szkuta B. DNA Transfer in Forensic Science: Recent Progress towards Meeting Challenges. Genes. 2021;12(11):1766.

[53]	Gill P, Fonnelop AE, Hicks T, Xenophontos S, Cariolou M, van Oorschot R, et al. The ReAct project: Analysis of data from 23 different laboratories to characterise DNA recovery given two sets of activity level propositions. Forensic Sci Int Genet. 2025;76:103222.

[54]	Gill P, Hicks T, Carracedo A. The ReAct project: Bayesian networks for assessing the value of the results given activity level propositions. Forensic Sci Int Genet. 2025;76:103223.

[55]	Stacey J, Opperman S, Curran JM, Harbison SA. Current state and barriers to global adoption of forensic evaluative reporting of findings given activity-level propositions. Science & Justice. 2025;65:101336.

[56]	Gill P, Hicks T, Kokshoorn B, van Oorschot RAH, Taylor D, Parson W. Minimum FSI: Genetics requirements for publishing data on DNA transfer and recovery, given activities. Forensic Sci Int Genet. 2026;80:103330.

[57]	Broderius K, Gosch A, Bamberg M, Foley MM, Kamphausen T, Onofri M, et al. Letter to the Editor Response to "Minimum FSI: Genetics requirements for publishing data on DNA transfer and recovery, given activities". Forensic Sci Int Genet. 2026;84:103507.

[58]	Kathrin Broderius, Iris Buckel, Galina Kulstein, Christa Augustin, Oliver Krebs, Janine Silvery, et al. Methods matter: On the impact of non-transferential variables on reported data of studies on nucleic acid transfer, prevalence, persistence and recovery (TPPR). Available at SSRN: <https://ssrn.com/abstract=7149758> or <http://dx.doi.org/10.2139/ssrn.7149758>.

[59]	Taylor D, Volgin L, Gill P, Kokshoorn B. Accounting for inter-laboratory DNA recovery data variability when performing evaluations given activities. Forensic Sci Int Genet. 2025;78:103283.

[60]	van Oorschot RAH, Szkuta B, Ballantyne KN, Goray M. Need for dedicated training, competency assessment, authorisations and ongoing proficiency testing for those addressing DNA transfer issues. Forensic Science International: Genetics Supplement Series. 2017;6:e32-e4.

[61]	Hicks T, Biedermann A, Taroni F, Champod C. Problematic reporting in DNA cases: the need for accredited formats and certified reporting competence. Forensic Science International: Genetics Supplement Series. 2019;7(1):205-7.

[62]	Gill P, Andersen MM, Whitaker JP, Kal AJ, Parson W. Birgitte Tengs case: analysis and the wider implications for evaluation of DNA evidence given activities. Forensic Science International: Genetics. 2025.

[63]	Dawid AP, Dotto F, Graves M, Kadane JB, Mortera J, Robertson G, et al. A comparison of graphical methods using the case of the murder of Meredith Kercher as an example. Law, Probability and Risk. 2025;24:1-26.

[64]	De March I, Taroni F. Bayesian networks and dissonant items of evidence: A case study. Forensic Sci Int Genet. 2020;44:102172.

[65]	Volgin L. The importance of evaluating findings given activity level propositions in order to avoid misleading evidence. Australian Journal of Forensic Sciences. 2019;51(sup1):S10-S3.

[66]	van Oorschot R, Ballantyne K, Mitchell RJ. Forensic trace DNA: a review. Investigative Genetics. 2010;1(1):14.

[67]	Meakin G, Jamieson A. DNA transfer: Review and implications for casework. Forensic Science International: Genetics. 2013;7(4):434-43.

[68]	van Oorschot RAH, Szkuta B, Meakin GE, Kokshoorn B, Goray M. DNA transfer in forensic science: A review. Forensic Science International: Genetics. 2019;38:140-66.

[69]	Burrill J, Daniel B, Frascione N. A review of trace "Touch DNA" deposits: Variability factors and an exploration of cellular composition. Forensic Sci Int Genet. 2019;39:8-18.

[70]	Sessa F, Pomara C, Esposito M, Grassi P, Cocimano G, Salerno M. Indirect DNA Transfer and Forensic Implications: A Literature Review. Genes. 2023;14(12):2153.

[71]	Woollacott C, Goray M, van Oorschot RAH, Taylor D. The Transfer, Prevalence, Persistence, and Recovery of DNA from Body Areas in Forensic Science: A Review. Forensic Sciences. 2025;5(1):9.

1. <https://www.iso.org/standard/72040.html> [accessed October 21, 2025]
2. <https://www.iso.org/standard/72039.html> [accessed October 21, 2025]
3. <https://www.iso.org/standard/73896.html> [accessed October 21, 2025]
